# An improved measure for belief structure in the evidence theory

**DOI:** 10.7717/peerj-cs.710

**Published:** 2021-09-24

**Authors:** Qiang Zhang, Hao Li, Rongfei Li, Yongchuan Tang

**Affiliations:** 1 School of Automation, Chongqing University, Chongqing, Chongqing, China; 2 School of Physics, Chongqing University, Chongqing, Chongqing, China; 3 School of Microelectronics and Communication Engineering, Chongqing University, Chongqing, Chongqing, China; 4 School of Big Data and Software Engineering, Chongqing University, Chongqing, China

**Keywords:** Evidence theory, Belief structure, Uncertainty measure, Euclidean distance, Classification

## Abstract

Dempster–Shafer evidence theory (D–S theory) is suitable for processing uncertain information under complex circumstances. However, how to measure the uncertainty of basic probability distribution (BPA) in D–S theory is still an open question. In this paper, a method of measuring total uncertainty based on belief interval distance is proposed. This method is directly defined in the D–S theoretical framework, without the need of converting BPA into probability distribution by Pignistic probability transformation. Thus, it avoids the loss of information. This paper analyzes the advantages and disadvantages of the previous total uncertainty of measurement, and the uncertainty measurement examples show the effectiveness of the new uncertainty measure. Finally, an information fusion method based on the new uncertainty measure is proposed. The validity and rationality of the proposed method are verified by two classification experiments from UCI data sets.

## Introduction

Uncertain information processing methods have always been a hot topic in the application of information fusion technology (Zhang & Mahadevan, 2019; He, Jiang & Chan, 2017). Dempster–Shafer evidence theory (D–S theory) (Shafer, 1976), as a typical tool for intelligent processing of uncertain information and information fusion, has been extensively studied. It has a wide range of applications in both military and civilian fields, such as target recognition (Li et al., 2016), classification (Xu et al., 2020; Liu et al., 2019; Tang, Wu & Liu, 2021; Wu, Liu & Tang, 2020), fault diagnosis (Fan & Zuo, 2006), risk analysis (Yang et al., 2011), decision-making (Fu, Chang & Yang, 2020; Fu et al., 2020) and pattern recognition (Zhou et al., 2018; Liu et al., 2020). D–S theory is an extension of probability theory, which in some cases can be degenerated into probability theory. In D–S theory, the Dempster’s combination rule is used to fuse basic probability assignment (BPA). However, when classical combination rules are used to fuse conflicting evidence, the result may be counterintuitive (Martin, 2019; Su et al., 2019). Therefore, in order to solve this problem, we can apply uncertainty measurement to the pre-processing before information fusion (Yong et al., 2004; Jing & Tang, 2021), so as to obtain more accurate information fusion results.

As the basis of information processing and evaluation, uncertainty plays an important role in the process of information processing. We can make use of the uncertainty of each information to preprocess the information appropriately to reduce the conflict or unreliability between the information. However, how to measure the uncertainty in the belief function is still an open question. In D–S theory, there are two types of uncertainty (Yager, 2008), namely conflict (Körner & Näther, 1995) and non-specificity (Dubois & Prade, 1985). In view of the above uncertainties, scholars have proposed different measurement methods for individual and total uncertainty (Jiroušek & Shenoy, 2020; Dubois & Prade, 1987). The measurement methods of conflict include the strife (Klir & Parviz, 1992) and the confusion (Hohle, 1982), etc. The measurement methods of non-specificity include Yager’s measure (Yager, 2008) and Hartley’s entropy-based (Hartley, 1928) measurement proposed by Dubois & Prade (1985). In addition, for the measurement methods of total uncertainty in the belief function, aggregated uncertainty (*AU*) (Harmanec & Klir, 1994) and ambiguity measure (*AM*) (Jousselme et al., 2006) are the most representative measurement methods. Although they meet the five axiom requirements proposed by Klir & Wierman (2013) and have been widely applied, they still have some shortcomings (Abellán & Masegosa, 2008; Klir & Lewis, 2008; Klir & Smith, 1999). For example, *AU*’s calculation is highly complex and insensitive to BPA changes. Although *AM* has ameliorated some of *AU*’s problems, it does not distinguish the uncertainty of different BPA with the same probability assignment. Recently, some new uncertainty measures in D–S theory are also proposed by researchers including the decomposable entropy in Jiroušek & Shenoy (2018, 2020), the Deng entropy (Deng, 2016), the correlation coefficient (Jiang, 2018) the divergence measure (Xiao, 2020) and so on (Song et al., 2018).

In general, the above uncertainty measurement methods are developed on the basis of Shannon entropy (Shannon, 2001). They need to convert basic probability assignment (BPA) into probability distribution under certain probability conversion rules (Smets & Kennes, 1994), and then calculate Shannon entropy to measure uncertainty. However, the conversion between such frameworks will lead to certain information loss, thus resulting in certain limitations. Therefore, in order to avoid this problem, a measurement method defined directly under the framework of D–S theory should be proposed (Yang & Han, 2016; Deng, Xiao & Deng, 2017).

Yang and Han proposed a new distance-based measure of total uncertainty (Yang & Han, 2016), which is a measure directly defined in the framework of D–S theory. They analyzed that the belief interval [*Bel*(*A*), *Pl*(*A*)] of the elements in the frame of discernment contained both conflict and non-specific uncertainties in the D–S theory, and the total uncertainty could be obtained by calculating the distance between the belief interval of each element and the maximum uncertainty case [*Bel*(*A*),*Pl*(*A*)] = [0, 1]. The magnitude of uncertainty is inversely proportional to the distance. However, Deng, Xiao & Deng’s (2017) research found that this method has some defects, which may lead to counter-intuitive results in some cases. In order to solve this problem, Deng et al. changed the calculation method of distance based on Yang & Han’s (2016) measure.

In this paper, we propose a new measure of total uncertainty noted as *ZU*, which is directly defined in the framework of D–S theory. This *ZU* can also solve the defects of Yang and Han’s measure mentioned above. This method gets total uncertainty by calculating the distance between the belief interval of each element and the interval [0.1], which avoids the limitation caused by the transformation of the framework in the calculation process, and the calculation method is relatively simple.

The rest of this paper are arranged as following. In “Basics of D–S Theory”, the related concepts of D–S theory are introduced. In “Analysis of Existing Measures”, various uncertainty measurement methods in D–S theory are introduced, and the limitation of partial total uncertainty measurement method is illustrated with numerical examples. In “Proposed Method”, a new total uncertainty measurement method *ZU* is proposed and its properties are analyzed. In “Numerical Example”, numerical examples are used to verify the effectiveness of the measurement method *ZU*. In “Application in Classification”, we propose a new classification method based on *ZU*, and verify its performance with two experiments. In “Conclusion”, the full paper is summarized.

## Basics of d–s theory

Some basic concepts of D–S theory are as follows.

The Frame of Discernment (FOD) is a non-empty set of information processed by D–S theory, defined as $\Omega = \left\{ {{h_1},{h_2}, \ldots ,{h_i}, \ldots ,{h_N}} \right\}$. The power set of the Frame of Discernment contains 2^*N*^ elements, which are expressed as follows:

(1) $${2^\Omega } = \left\{ {\emptyset ,\left\{ {{h_1}} \right\}, \ldots ,\left\{ {{h_N}} \right\},\left\{ {{h_1},{h_2}} \right\}, \ldots ,\left\{ {{h_1},{h_2}, \ldots ,{h_i}} \right\}, \ldots ,\Omega } \right\}.$$

A mass function *m* (BPA) is the mapping of the power set of the FOD on the interval [0, 1], which satisfies the following relation:

(2) $$m(\emptyset ) = 0,\sum\limits_{A \in {2^\Omega }} m(A) = 1.$$

The mass function can also be expressed by the belief function (*Bel*) and the plausibility function (*Pl*), defined as follows (Smets & Kennes, 1994):

(3) $$Bel(A) = \sum\limits_{\emptyset \ne B \subseteq A} m(B),Pl(A) = \sum\limits_{B \cap A \ne \emptyset } m(B)$$

The *Bel*(*A*) represents the lower limit value of evidence’s support for proposition *A*. And the *Pl*(*A*) represents the upper limit value of evidence’s support for this proposition.

In the D–S theory, two groups of independent mass functions *m*_1_ and *m*_2_ can conduct data fusion through Dempster’s rule, which satisfies:

(4) $$m(A) = \left( {{m_1} \oplus {m_2}} \right)(A) = \displaystyle{1 \over {1 - k}}\sum\limits_{B \cap C = A} {m_1}(B){m_2}(C),$$

where, *k* is a normalized factor, defined as follows:

$$k = \sum\limits_{B \cap C = \emptyset } {m_1}(B){m_2}(C).$$

For subset *A*, its mass function can be converted to probability distribution by Pignistic probability conversion. The transformation to meet mapping *BetP*_*m*_ : $\Omega \to [0,1]$, is defined as follows:

(5) $$Bet{P_m}\left( {{A_i}} \right) = \sum\limits_{A \subset P(\Omega ),{A_i} \in A} \displaystyle{{m(A)} \over {|A|}},$$

where, $\left| A \right|$ represents the cardinality of subset *A*.

## Analysis of existing measures

The total uncertainty measure is to simultaneously measure the two kinds of uncertainty, namely conflict and non-specificity. And the aggregated uncertainty (*AU*) and ambiguity measure (*AM*) are the most representative measurement methods.

### Aggregated uncertainty and ambiguity measure

The Aggregated uncertainty (*AU*) is defined as (Harmanec & Klir, 1994):

(6) $$AU(m) = \max \left[ { - \sum\limits_{h \in \Omega } {p_h}\mathop {\log }\nolimits_2 {p_h}} \right]$$

$$s.t.\left\{ {\matrix{ {{p_h} \in [0,1],\forall h \in \Omega } \hfill \cr {Bel(A) \le \sum\limits_{h \in A} {p_h} \le 1 - Bel(\bar A),\forall A \subseteq \Omega } \hfill \cr {\sum\limits_{h \in \Omega } {p_h} = 1} \hfill \cr } } \right.$$

*AU* refers to the value of the maximum Shannon entropy corresponding to the given mass function. Therefore, it is also called “upper entropy”.

The ambiguity measure (*AM*) is defined as (Jousselme et al., 2006):

(7) $$AM(m) = - \sum\limits_{h \in \Omega } Bet{P_m}(h)\mathop {\log }\nolimits_2 \left( {Bet{P_m}(h)} \right)$$

*AM* converts BPA into probability distribution by Pignistic probabilistic conversion, and then the total uncertainty is calculated. However, *AU* and *AM* have some limitations, which will be analyzed by the following example 1.

**Example 1** Define that FOD $\Omega = \left\{ {{h_1},{h_2},{h_3},{h_4}} \right\}$, three BPAs are given as:

$$\matrix{ {{m_1}(\Omega ) = 1} \hfill \cr {{m_2}\left( {{h_1},{h_2}} \right) = \displaystyle{1 \over 2},{m_2}\left( {{h_3},{h_4}} \right) = \displaystyle{1 \over 2}} \hfill \cr {{m_3}\left( {{h_1}} \right) = {m_3}\left( {{h_2}} \right) = {m_3}\left( {{h_3}} \right) = {m_3}\left( {{h_4}} \right) = \displaystyle{1 \over 4}} \hfill \cr }$$

Evidently, we can find that for *m*_1_, The BPA: *m*_1_(Ω) = 1 represents total ignorance. For *m*_2_, it divided the elements into groups of two and divided the probabilities equally among the groups, while *m*_3_ divided the probabilities equally among each element. Therefore, it is intuitive that the uncertainty of the three mass functions should gradually decrease. However, the calculation results of *AU* and *AM* are contrary to the intuitive results, which are shown as follows:

$$\matrix{ {AU\left( {{m_1}} \right) = AU\left( {{m_2}} \right) = AU\left( {{m_3}} \right) = 2} \hfill \cr {AM\left( {{m_1}} \right) = AM\left( {{m_2}} \right) = AM\left( {{m_3}} \right) = 2} \hfill \cr }$$

We can observe that the value of all three BPA, *AU* and *AM* is 2, which is obviously against the intuition. The reason for this is as follows: For *AU*, it focuses on the maximum Shannon entropy for the given BPA condition. So it’s not sensitive to changes in BPA. For *AM*, we can calculate that by applying Pignistic probability conversion to each BPA, the three BPA have the same probability distribution, that is $Bet{P_m}\left( {{h_i}} \right) = 0.25,(i = 1,2,3,4)$, so the calculation results of *AM* are unchanged.

### The total uncertainty measure *TU*^*I*^

Define that FOD $\Omega = \left\{ {{h_1},{h_2}, \cdots ,{h_i}, \cdots ,{h_N}} \right\}$, the total uncertainty measure *TU*^*I*^ by Yang and Han’s is defined as (Yang & Han, 2016):

(8) $$T{U^I}(m) = 1 - \displaystyle{1 \over n} \cdot \sqrt 3 \cdot \sum\limits_{i = 1}^n {d^I}\left( {\left[ {Bel\left( {{h_i}} \right),Pl\left( {{h_i}} \right)} \right],[0,1]} \right).$$

with

(9) $$\matrix{ {{d^I}\left( {\left[ {{a_1},{b_1}} \right],\left[ {{a_2},{b_2}} \right]} \right) = \sqrt {{{\left[ {\displaystyle{{{a_1} + {b_1}} \over 2} - \displaystyle{{{a_2} + {b_2}} \over 2}} \right]}^2} + \displaystyle{1 \over 3}{{\left[ {\displaystyle{{{b_1} - {a_1}} \over 2} - \displaystyle{{{b_2} - {a_2}} \over 2}} \right]}^2}} .} \hfill \cr }$$

*TU*^*I*^ obtains the total uncertainty by calculating the distance between the belief interval of each element and the interval [0.1], and finally normalized the value of the total uncertainty range into [0,1]. However, although *TU*^*I*^ is sensitive to BPA changes and has low computational complexity, counter-intuitive results can occur under certain circumstances, which will be analyzed by the following example 2.

**Example 2** Define that FOD $\Omega = \left\{ {{h_1},{h_2}} \right\}$, two BPAs are given as:

$$\matrix{ {{m_1}\left( {{h_1}} \right) = \displaystyle{1 \over 2},{m_1}\left( {{h_2}} \right) = \displaystyle{1 \over 2}.{m_2}\left( {{h_2}} \right) = \displaystyle{1 \over 2},{m_2}(\Omega ) = \displaystyle{1 \over 2}.} \hfill \cr }$$

For the two BPA groups, the belief intervals are as follows:

$$\matrix{ {} \hfill  {\left\{ {\matrix{ {{\vskip10pt}\left[ {Be{l_1}\left( {{h_1}} \right),P{l_1}\left( {{h_1}} \right)} \right] = \left[\displaystyle{1 \over 2},\displaystyle{1 \over 2} \right ]} \hfill \cr {\left[ {Be{l_1}\left( {{h_2}} \right),P{l_1}\left( {{h_2}} \right)} \right] = \left[\displaystyle{1 \over 2},\displaystyle{1 \over 2} \right]} \hfill \cr {\left[ {Be{l_1}(\Omega ),P{l_1}(\Omega )} \right] = [1,1]} \hfill \cr } } \right.} \hfill \cr {} \hfill  {\left\{ {\matrix{ {\left[ {Be{l_2}\left( {{h_1}} \right),P{l_2}\left( {{h_1}} \right)} \right] = \left[0,\displaystyle{1 \over 2} \right]} \hfill \cr {\left[ {Be{l_2}\left( {{h_2}} \right),P{l_2}\left( {{h_2}} \right)} \right] = \left[\displaystyle{1 \over 2},1 \right]} \hfill \cr {\left[ {Be{l_2}(\Omega ),P{l_2}(\Omega )} \right] = [1,1]} \hfill \cr } } \right.} \hfill \cr }$$

By comparing the belief intervals of *m*_1_ and *m*_2_, we can get that ∀ *A* ∈ Ω, $\left[ {Be{l_1}(A),P{l_1}(A)} \right] \subseteq \left[ {Be{l_2}(A),P{l_2}(A)} \right]$, which means that the belief intervals of *m*_1_ are all proper subsets of the belief intervals of *m*_2_. Therefore, the uncertainty of *m*_2_ should be higher than *m*_1_. Here are the results of several measurements.

$$\matrix{ {AU\left( {{m_1}} \right) = AU\left( {{m_2}} \right) = 1} \hfill \cr {AM\left( {{m_1}} \right) = 1,AM\left( {{m_2}} \right) = 0.8113} \hfill \cr {T{U^I}\left( {{m_1}} \right) = T{U^I}\left( {{m_2}} \right) = \displaystyle{1 \over 2}} \hfill \cr }$$

We can observe that the results of the three measures appear counterintuitive, and *AM* appears contrary to monotonicity. Therefore, in this case, none of the three measures can correctly quantify the total uncertainty.

Through the above description, we can observe that there are some limitations in the application of the above total uncertainty measurement methods. Therefore, in the next section, we will propose a new measure of total uncertainty, called *ZU*, which is directly defined in the framework of D–S theory. The *ZU* can solve the defects of Yang and Han’s measure mentioned above.

## Proposed method

### The new total uncertainty measure

In the belief interval of focal element *A*, the information related to uncertainty includes the conflict part and the non-specificity part (Yang & Han, 2016). Therefore, for a focal element *A*, its belief interval can represent its degree of uncertainty, and its value can be expressed by the Euclidean distance between the belief interval of *A* and the most uncertain case [*Bel*(*A*),*Pl*(*A*)] = [0,1], as shown in the following formula:

(10) $$d([Bel(A),Pl(A)],[0,1]) = \sqrt {{{\left[ {Bel(A) - 0} \right]}^2} + {{\left[ {Pl(A) - 1} \right]}^2}}$$

When [*Bel*(*A*),*Pl*(*A*)] = [0,1], it means complete unknown, with the greatest degree of uncertainty. Therefore, when the belief interval distance of *A* is far from the most uncertain case [0,1], the distance value is large and indicates that the uncertainty is small; conversely, the smaller the value is, the greater the degree of uncertainty is. Therefore, for a given BPA, the total uncertainty can be expressed by calculating the distance between the belief interval of each element and the interval [0,1], and then integrating it through the following formula:

(11) $$ZU(m) = \sum\limits_{i = 1}^n \displaystyle{4 \over 3}\left( {\displaystyle{1 \over {{{(1 + d)}^2}}} - \displaystyle{1 \over 4}} \right)$$

In the above formula, *n* is the cardinality of FOD Ω, and the formula expresses the decreasing relationship between distance *d* and uncertainty through the inverse proportional relationship. After trying a variety of functional expressions, we choose 1/(1 + *d*)^2^ with better experimental effect as the main part of the formula. In addition, the selection of 4/3 and 1/4 parameters is to limit that the value range of the uncertainty for any element is [0, 1]. That is, when *d* = 0, the value of the uncertainty is 1. And when *d* = 1, the value is 0, so its property of boundedness is guaranteed.

**Figure 1:**
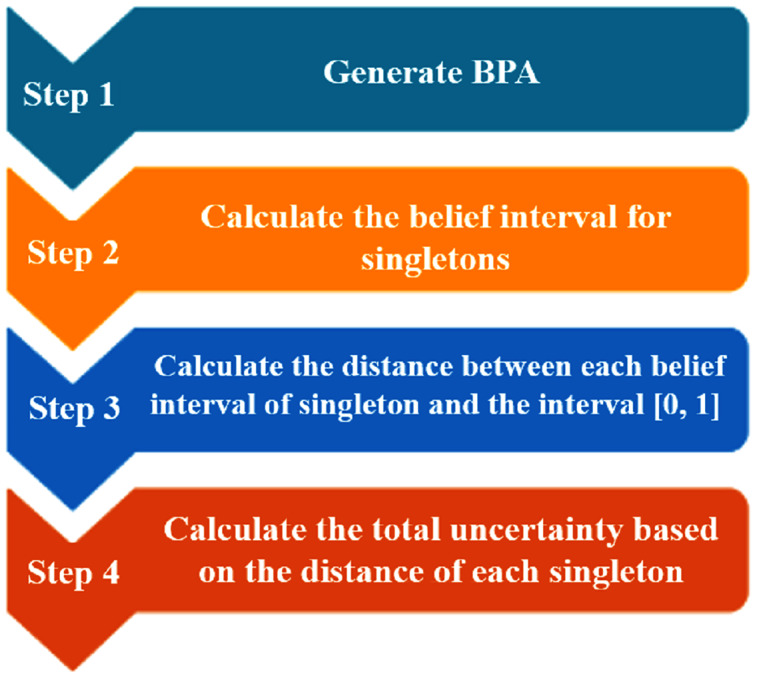
The flowchart of generating the total uncertainty measurement.

The steps of calculating the total uncertainty by *ZU* are shown in Fig. 1. And to demonstrate how to calculate *ZU*, we provide an example as follows:

Define that FOD $\Omega = \left\{ {{h_1},{h_2},{h_3}} \right\}$, the BPA is given as: $m\left( {{h_1}} \right) = 0.2,$ $m\left( {{h_1},{h_2}} \right) = 0.5,$ $m\left( {{h_2},{h_3}} \right) = 0.3$. The specific calculation process of its total uncertainty is shown below:

Step 1: Calculate the belief interval for singletons

$$\left\{ {\matrix{ {Bel\left( {{h_1}} \right) = m\left( {{h_1}} \right) = 0.2} \hfill \cr {Bel\left( {{h_2}} \right) = m\left( {{h_2}} \right) = 0} \hfill \cr {Bel\left( {{h_3}} \right) = m\left( {{h_3}} \right) = 0} \hfill \cr {Pl\left( {{h_1}} \right) = m\left( {{h_1}} \right) + m\left( {{h_1},{h_2}} \right) = 0.7} \hfill \cr {Pl\left( {{h_2}} \right) = m\left( {{h_2}} \right) + m\left( {{h_1},{h_2}} \right) + m\left( {{h_2},{h_3}} \right) = 0.8} \hfill \cr {Pl\left( {{h_3}} \right) = m\left( {{h_3}} \right) + m\left( {{h_2},{h_3}} \right) = 0.3} \hfill \cr } } \right.$$

Step 2: Calculate the distance between each belief interval of singleton and the interval [0, 1]

$$ \matrix{ \hfill \cr {d\left( {\left[ {Bel\left( {{h_3}} \right),Pl\left( {{h_3}} \right)} \right],[0,1]} \right)} \hfill  { = d([0,0.3],[0,1])} \hfill \cr {} \hfill  { = \sqrt {{{(0 - 0)}^2} + {{(0.3 - 1)}^2}} } \hfill \cr {} \hfill  { = 0.7} \hfill \cr }$$

Step 3: Calculate the total uncertainty based on the distance of each singleton

$$\matrix{ {ZU(m)} \hfill  { = \sum\limits_{i = 1}^n \displaystyle{4 \over 3}\left( {\displaystyle{1 \over {{{(1 + d)}^2}}} - \displaystyle{1 \over 4}} \right)} \hfill \cr {} \hfill  { = \displaystyle{4 \over 3}\left( {\displaystyle{1 \over {{{(1 + 0.36)}^2}}} - \displaystyle{1 \over 4}} \right) + \displaystyle{4 \over 3}\left( {\displaystyle{1 \over {{{(1 + 0.2)}^2}}} - \displaystyle{1 \over 4}} \right)} \hfill \cr {} \hfill  { + \displaystyle{4 \over 3}\left( {\displaystyle{1 \over {{{(1 + 0.7)}^2}}} - \displaystyle{1 \over 4}} \right) = 1.10816} \hfill \cr }$$

### Some properties of the proposed uncertainty measure

**Property 1: monotonicity** Assuming that there are two groups of BPA *m*_1_ and *m*_2_, which are defined on FOD Ω. If $\left[ {Be{l_1}(A),P{l_1}(A)} \right] \subseteq \left[ {Be{l_2}(A),P{l_2}(A)} \right]$, *∀ A* ∈ Ω, then *ZU*(*m*_1_) ≤ *ZU*(*m*_2_).

**Proof of Monotonicity:** By the calculation formula of distance, if $\left[ {Be{l_1}(A),P{l_1}(A)} \right] \subseteq \left[ {Be{l_2}(A),P{l_2}(A)} \right]$, ∀ *A* ∈ Ω, then $d\left( {\left[ {Be{l_1}(A),P{l_1}(A)} \right]} \right) \ge d\left( {\left[ {Be{l_2}(A),P{l_2}(A)} \right]} \right)$, *∀ A* ∈ Ω. In addition, through the final integration formula, it can be seen that the value of *ZU*(*m*) is inversely proportional to the value of distance, that is, the larger distance is, the smaller *ZU* (*m*) is. So we can get *ZU* (*m*_1_) ≤ *ZU* (*m*_2_).

**Property 2: boundness** The value range of *ZU*(*m*) is [0,*N*], where N is the cardinality of FOD Ω.

**Proof of Boundness:** when the BPA is a vacuous BPA, that is *m*(Ω) = 1, we have [*Bel*(*A*),*Pl*(*A*)] = [0,1], *∀ A* ∈ Ω. In this case $d\left( {[Bel(A),Pl(A)],[0,1]} \right) = 0$, *∀ A* ∈ Ω. And through the final integration formula, the result is obtained to be *ZU*(*m*) = *N*. In addition, since the belief interval of any BPA is a subset of [0,1], it can be known from the monotony obtained above that N is the maximum value of *ZU* (*m*). Similarly, the lower bound of *ZU*(*m*) is 0. This result is obtained when $d\left( {[Bel(A),Pl(A)],[0,1]} \right) = 1$, *∀ A* ∈ Ω, which corresponds to [*Bel*(*A*),*Pl*(*A*)] = [0,0] or [1,1], *∀ A* ∈ Ω. This is a completely accurate case, where the uncertainty is zero.

**Property 3: invariance** Assuming that *m* is a BPA defined in FOD Ω, the total uncertainty of *m* is represented as $Z{U_\Omega }\left( m \right)$, then $Z{U_\Omega }\left( m \right) = Z{U_\Theta }\left( m \right)$, where Θ = Ω ∪ *ϕ* and *ϕ* is an arbitrary set.

**Proof of Invariance:** Assuming that each singleton of Θ is *h*_*i*_, Since BPA *m* is initially defined on Ω, we can get $\left[ {Bel\left( {{h_i}} \right),Pl\left( {{h_i}} \right)} \right] = \left[ {0,0} \right]$, when *h*_*i*_ ∈ Φ and *h*_*i*_ *∉ Ω*. Thus,

$$\eqalign{ \matrix{ {Z{U_\Theta }(m)} = {\sum\limits_{{h_i} \in \Omega } \left[ {\displaystyle{4 \over 3}\left( {\displaystyle{1 \over {{{\left( {1 + d\left( {\left[ {{\rm Bel}\left( {{h_i}} \right),Pl\left( {{h_i}} \right)} \right],[0,1]} \right)} \right)}^2}}} - \displaystyle{1 \over 4}} \right)} \right]} \cr { + \sum\limits_{{h_i} \notin \Omega ,{h_i} \in \Phi } \left[ {\displaystyle{4 \over 3}\left( {\displaystyle{1 \over {{{\left( {1 + d([0,0],[0,1])} \right)}^2}}} - \displaystyle{1 \over 4}} \right)} \right]}} \cr\qquad\quad = {{\sum\limits_{{h_i} \in \Omega } \left[ {\displaystyle{4 \over 3}\left( {\displaystyle{1 \over {{{\left( {1 + d\left( {\left[ {{\rm Bel}\left( {{h_i}} \right),Pl\left( {{h_i}} \right)} \right],[0,1]} \right)} \right)}^2}}} - \displaystyle{1 \over 4}} \right)} \right] + \sum\limits_{{h_i} \notin \Omega ,{h_i} \in \Phi } 0}} \cr\qquad\quad = {{\sum\limits_{{h_i} \in \Omega } \left[ {\displaystyle{4 \over 3}\left( {\displaystyle{1 \over {{{\left( {1 + d\left( {\left[ {{\rm Bel}\left( {{h_i}} \right),Pl\left( {{h_i}} \right)} \right],[0,1]} \right)} \right)}^2}}} - \displaystyle{1 \over 4}} \right)} \right] = Z{U_\Omega }(m)} \hfill \cr {} \hfill  {} \hfill \cr } }$$

**Property 4: monotonicity among Bayesian BPAs** Bayesian BPA is defined as $\sum\nolimits_{A \subseteq \Omega } m(A) = 1$ and $m\left( A \right) = 0,\forall \left| A \right| \ne 1$. For the Bayesian BPA, it has the following properties:

**Property 4.1**: Suppose *m* is the Bayesian BPA, defined as FOD $\Omega = \left\{ {{h_{1,}}{h_2},...,{h_n}} \right\},n\ {\ge }\ 2$. When the $m\left( {{h_i}} \right) = \textstyle{1 \over n},i = 1,2,...,n$, *ZU*(*m*) obtains the maximum value.

**Proof of Property 4.1:** When $m\left( A \right) = 0,\forall \left| A \right| \ne 1$, we can get $m\left( {{h_i}} \right) = Bel\left( {{h_i}} \right) = Pl\left( {{h_i}} \right)$. Therefore, we can assume that $m\left( {{h_i}} \right) = Bel\left( {{h_i}} \right) = Pl\left( {{h_i}} \right) = {a_i},\forall {h_i} \in \Omega$, and $\sum\nolimits_{i = 1}^n {a_i} = 1$.

So the distance is:

$$\matrix{ {d([Bel(A),Pl(A)],[0,1])} \hfill & { = \sqrt {{{\left( {{a_i} - 0} \right)}^2} + {{\left( {{a_i} - 1} \right)}^2}} } \hfill \cr {} \hfill & { = \sqrt {2{a_i}^2 - 2{a_i} + 1} } \hfill \cr }$$

To obtain the maximum value of *ZU*(*m*), the Lagrange function can be obtained as follows:

$$\matrix{ {L\left( m \right) = \sum\limits_{i = 1}^n \displaystyle{4 \over 3}\left( {\displaystyle{1 \over {{{\left( {1 + \sqrt {2a_i^2 - 2{a_i} + 1} } \right)}^2}}} - \displaystyle{1 \over 4}} \right) + \lambda \left( {\sum\limits_{i = 1}^n {a_i} - 1} \right)} \hfill \cr }$$

By taking the partial derivatives of the above function, we can get

$$\left\{ {\matrix{ {\displaystyle{{\partial L(m)} \over {\partial {a_i}}} = \displaystyle{4 \over 3}\left( {\displaystyle{{1 - 2{a_i}} \over {{{\left( {1 + \sqrt {2a_i^2 - 2{a_i} + 1} } \right)}^3} \times \sqrt {2a_i^2 - 2{a_i} + 1} }}} \right) + \lambda = 0} \hfill \cr {\displaystyle{{\partial L(m)} \over {\partial \lambda }} = \sum\limits_{i = 1}^n {a_i} - 1 = 0} \hfill \cr } } \right.$$

Assume that the numerator of $\textstyle{{\partial L\left( m \right)} \over {\partial {a_i}}}$ is ${N_1}\left( {{a_i}} \right)$, and the denominator is ${N_2}\left( {{a_i}} \right)$, where

$$N_2^{\prime}\left( {{a_i}} \right) = \displaystyle{3 \over 2}{\left( {1 + \sqrt {2a_i^2 - 2{a_i} + 1} } \right)^2} + \displaystyle{{{{\left( {1 + \sqrt {2a_i^2 - 2{a_i} + 1} } \right)}^3}} \over {2\sqrt {2a_i^2 - 2{a_i} + 1} }}$$

When ${a_i} \in \left[ {0,1} \right]$, we have $N_2^{\prime}\left( {{a_i}} \right) > 0$, so the denominator ${N_2}\left( {{a_i}} \right)$ increases monotonically at ${a_i} \in \left[ {0,1} \right]$, and ${N_2}\left( 0 \right) = 10$. For the numerator ${N_1}\left( {{a_i}} \right) = 1 - 2{a_i}$, it decreases monotonically and greater than 0 at ${a_i} \in \left[ {0,1} \right]$. Therefore, $\textstyle{{\partial L\left( m \right)} \over {\partial {a_i}}}$ is monotonically decreasing.

So the equation $\textstyle{{\partial L\left( m \right)} \over {\partial {a_i}}} = - \lambda$ has only one result, *a*_*i*_ = *x*,*i* = 1,2,…,*n*. Thus it can be obtained:

$$\left\{ {\matrix{ {{a_1} = {a_2} = ... = {a_n} = x} \cr {\sum\limits_{i = 1}^n {a_i} = 1} \right.$$

Therefore when the $m\left( {{h_i}} \right) = \displaystyle{1 \over n},i = 1,2,...,n$, *ZU*(*m*) obtains the maximum value.

**Property 4.2**: Suppose *m* is the Bayesian BPA, defined as FOD Ω, and $\left| \Omega \right| = n$. As *n* increases, its maximum uncertainty $\max ZU\left( m \right)$ increases.

**Proof of Property 4.2:** According to the derivation of Property 4.1, the maximum uncertainty $\max ZU\left( m \right)$ can be obtained by the following formula:

$$\matrix{ {f = \max ZU(m)} \hfill  { = \sum\limits_{i = 1}^n \displaystyle{4 \over 3}\left( {\displaystyle{1 \over {{{\left( {1 + \sqrt {2(1/n{)^2} - 2(1/n) + 1} } \right)}^2}}} - \displaystyle{1 \over 4}} \right)} \hfill \cr {} \hfill  { = \displaystyle{2 \over 3}\left( {\displaystyle{{{n^2}} \over {n - 1 + \displaystyle{1 \over n} + \sqrt {{n^2} - 2n + 2} }} - \displaystyle{1 \over 2}} \right)} \hfill \cr }$$

and

$$\matrix{ {{f^{\prime}}} \hfill  { = \displaystyle{2 \over 3} \times \displaystyle{{2n\left( {n - 1 + \displaystyle{1 \over n} + \sqrt {{n^2} - 2n + 2} } \right) - {n^2}\left( {1 - \displaystyle{1 \over {{n^2}}} + \displaystyle{{n - 1} \over {\sqrt {{n^2} - 2n + 2} }}} \right)} \over {{{\left( {n - 1 + \displaystyle{1 \over n} + \sqrt {{n^2} - 2n + 2} } \right)}^2}}}} \hfill \cr {} \hfill  { = \displaystyle{2 \over 3} \times \displaystyle{{{n^2} - 2n + 3 + \displaystyle{{{n^3} - 3{n^2} + 4n} \over {\sqrt {{n^2} - 2n + 2} }}} \over {{{\left( {n - 1 + \displaystyle{1 \over n} + \sqrt {{n^2} - 2n + 2} } \right)}^2}}}} \hfill \cr }$$

When *n* > 1, the numerator and denominator of the function *f*′ are both greater than 0, so *f*′ is greater than 0. Therefore, as *n* increases, $\max ZU\left( m \right)$ increases, conforming to the monotonicity.

**Property 5: sensitivity** *ZU* is sensitive to the changes of BPAs. Sensitivity refers to the relative change degree of total uncertainty when BPA changes. For *AU*, its variation range is $[0,\mathop {\log }\nolimits_2 \left| \Omega \right|]$, and in some cases, with the change of BPA, its total uncertainty will remain approximately unchanged. Therefore, its sensitivity is low. For *AM* and *TU*^*I*^, there are fewer restrictions on their use, but their value ranges are relatively small: $[0,\mathop {\log }\nolimits_2 \left| \Omega \right|]$ and [0, 1] respectively. When BPA changes, the obtained total uncertainty changes relatively little, so the sensitivity is moderate. For *ZU*, the value range is large, which is [0, *N*]. When BPA changes, its total uncertainty *ZU*(*m*) changes the most and its sensitivity is the highest. This property will also be illustrated with some numerical examples in the next section. And the comparison of properties of *AU*, *AM*, *TU*^*I*^ and *ZU* is shown in Table 1:

**Table 1:** Comparison of properties of each measurement method.

Measure	Boundness	Monotonicity	Invariance	Sensitivity
*AU*(*m*)	$[0,\mathop {\log }\nolimits_2 \left| \Omega \right|]$	*Satisfied*	*Satisfied*	*Low*
*AM*(*m*)	$[0,\mathop {\log }\nolimits_2 \left| \Omega \right|]$	×	*Satisfied*	*Medium*
*TU*^*I*^(*m*)	[0,1]	*Satisfied*	×	*Medium*
*ZU*(*m*)	[0,*N*]	*Satisfied*	*Satisfied*	*High*

## Numerical example

In this section, we will show several numerical examples to verify the performance of *ZU*, with the *AU*, *AM* and *TU*^*I*^ in comparison with the results of three methods of measurement.

**Continue to Example 1** Under the given BPAs in Example 1, the total uncertainty of the three groups of BPAs can be obtained through the calculation method of *ZU*(*m*) as follows: *ZU*(*m*_1_) = 4, *ZU*(*m*_2_) = 1.037, *ZU*(*m*_3_) = 0.33. It can be found that the uncertainty of the three groups of BPA in this result is gradually decreasing, which is consistent with the above analysis.

**Continue to Example 2** In the given two groups of BPA in Example 2, the belief interval of *m*_1_ is a true subset of the belief interval of *m*_2_ through the previous analysis. Therefore, the uncertainty of *m*_2_ should be higher than that of *m*_1_. Through the calculation method of *ZU* (*m*), the total uncertainty of the two groups of BPA can be obtained as follows: *ZU*(*m*_1_) = 0.2484, *ZU*(*m*_2_) = 0.5185. We can find that the result of *ZU* (*m*) is consistent with the actual analysis. Therefore, in this kind of problem, *ZU* ’s sensitivity to the BPA changes relative to *AU*, *AM* and *TU*^*I*^ is better.

**Figure 2:**
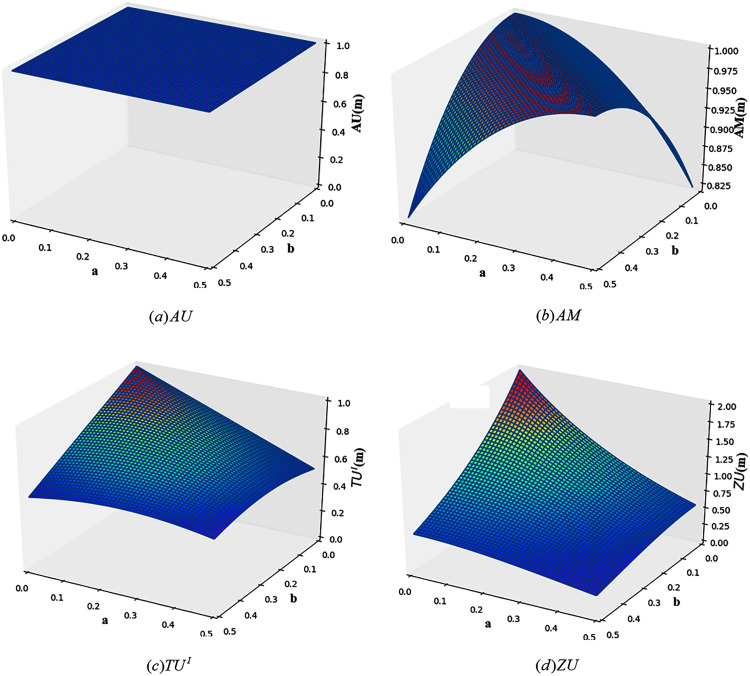
The change of total uncertainty measures in Example 3.

**Example 3** Define that FOD $\Omega = \left\{ {{h_1},{h_2},{h_3}} \right\}$, the BPA is given as: $m\left( {{h_1}} \right) = a$, $m\left( {{h_2}} \right) = b$, *m*(Ω) = 1 − *a* − *b*. Figure 2 shows the change images of total uncertainty obtained by four measurement methods, *AU*, *AM*, *TU*^*I*^ and *ZU*, under different values of *a* and *b*.

As can be seen from Fig. 2, the calculation results of *AU* and *AM* are both counterintuitive. For *AU*, with the change of *a* and *b*, the total uncertainty calculated from *AU* is always 1. For *AM*, when *a* = *b*, the total uncertainty does not change with the change of *a* and *b*, and is always the maximum. This is because when *a* = *b*, the probability is evenly distributed over the two elements, that is, *BetP*_*m*_(*h*_1_) = *BetP*_*m*_(*h*_2_) = 0.5. For *TU*^*I*^, it can be seen from the figure that when *a* and *b* change, the change of total uncertainty can be better reflected. But it doesn’t reflect the difference between *m*(*h*_1_) = *m*(*h*_2_) = 0.5 and *m*(*h*_1_) = *m*(Ω) = 0.5 or *m*(*h*_2_) = *m*(Ω) = 0.5. As shown in Fig. 2, *ZU* ’s measurement results are more reasonable. It is not only sensitive to the change of BPA, but also overcomes the problems of *TU*^*I*^.

**Figure 3:**
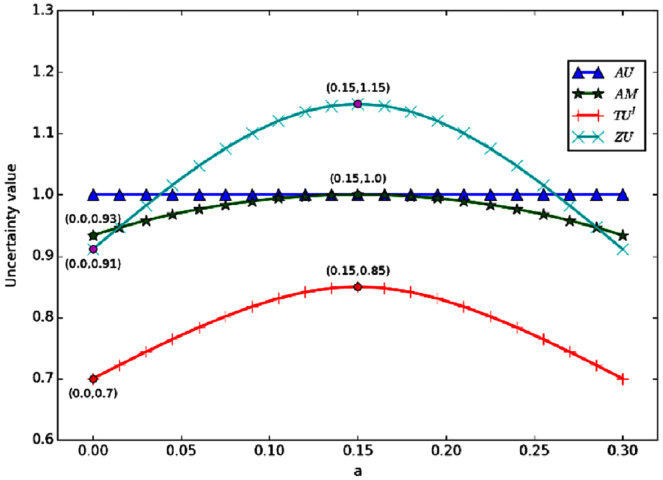
The change of when *a + b* = 0.3.

And from Fig. 3, we can find that when *a* + *b* is a certain value. If *a* = *b*, then the uncertainty reaches the maximum. The figure shows the change curve of uncertainty obtained by each measurement method with the change of *a* and *b*, when *a* + *b* = 0.3. According to the figure, when *a* = *b*, the uncertainty is the maximum. This is because, in the process of change, *m*(Ω) is a certain value of 0.7, which means that the non-specificity in the uncertainty is fixed. When *a* = *b*, the conflict is at its maximum. So the total uncertainty is the highest.

**Figure 4:**
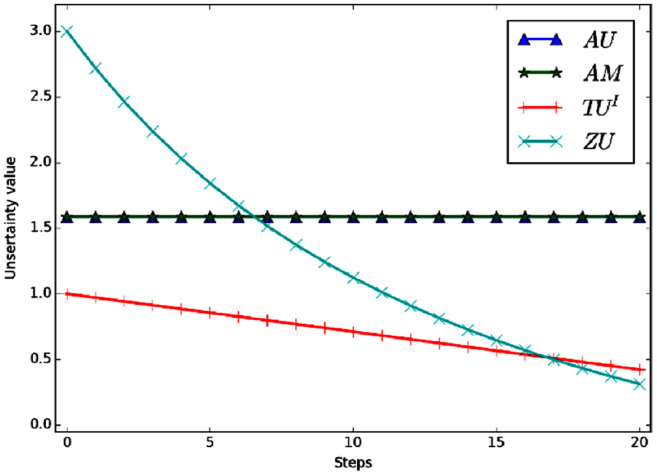
The change of total uncertainty measures in Example 4.

**Example 4** Define that FOD $\Omega = \left\{ {{h_1},{h_2},{h_3}} \right\}$, the BPA is *m*(Ω) = 1. Now let the BPA changes according to certain rule. In each step, *m*(Ω) decrease *Δ* = 0.05 and the mass of each singleton *m*(*h*_*i*_), *i* = 1, 2, 3 increase $\textstyle{\Delta \over 3}$. And the BPA will eventually become $m({h_1}) = m({h_2}) = m({h_3}) = \textstyle{1 \over 3}$. Figure 4 shows the variation curve of the total uncertainty obtained by each measurement method under the above conditions.

Evidently, the mass of *m*(Ω) shifted to singletons gradually in the process of change, So the total uncertainty should be decreasing continually. But in the figure we can see that *AU* and *AM* are a constant value, which is counterintuitive. The reason is that *AU* and *AM* need to carry out probability conversion, and the result after conversion is always $Bet{P_m}({h_i}) = \textstyle{1 \over 3}$, *i* = 1, 2, 3. So the values of *AU* and *AM* are always the same. *ZU* and *TU*^*I*^ can better reflect the change of total uncertainty. In the process of change, *TU*^*I*^ is a linear change and *ZU* is a nonlinear change. Moreover, *ZU* has a wider range of variation and higher sensitivity.

**Figure 5:**
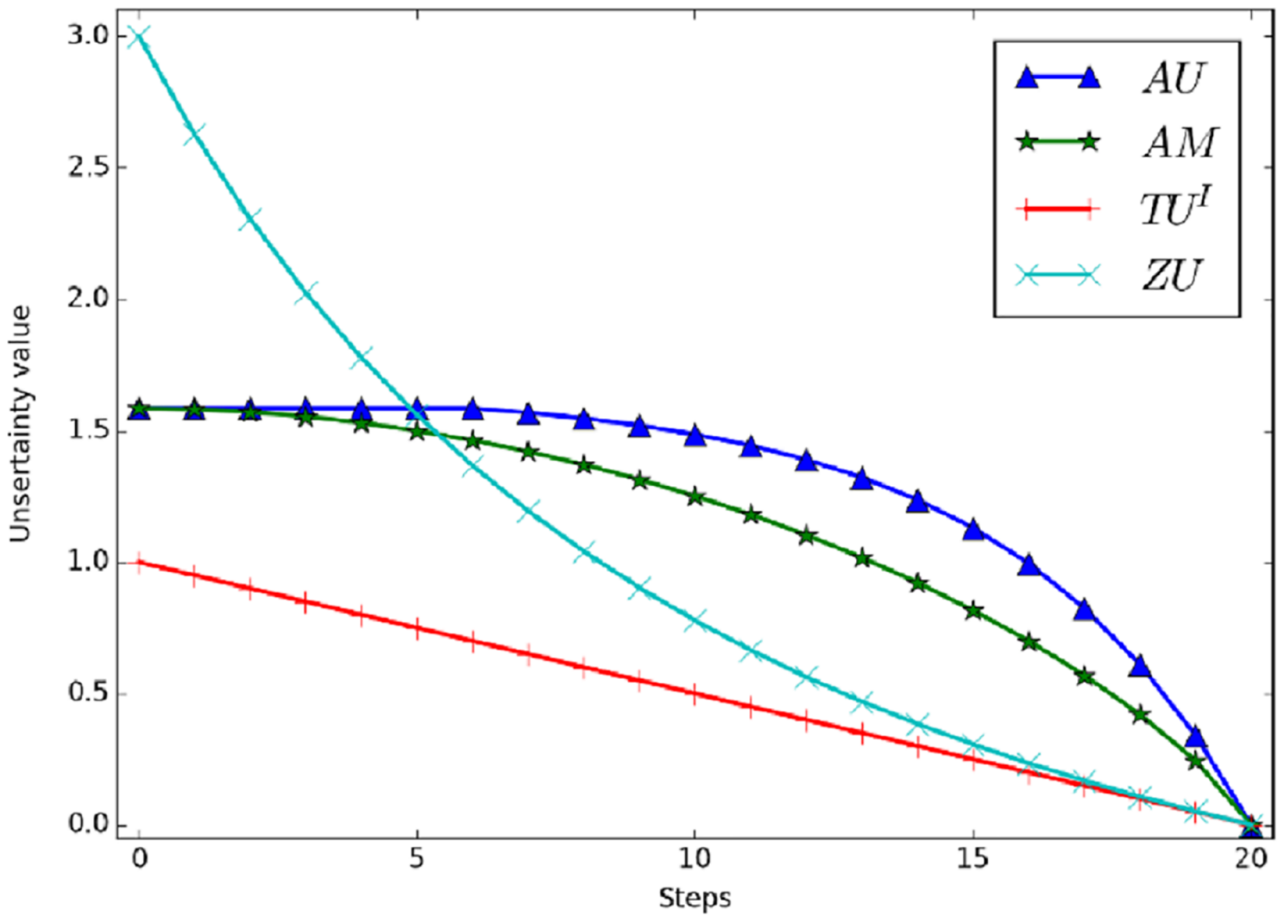
The total uncertainty measures in Example 5.

**Example 5** Define that FOD $\Omega = \left\{ {{h_1},{h_2},{h_3}} \right\}$, the initial given BPA is *m*(Ω) = 1. Now let the BPA changes with some regularity. In each step, *m*(Ω) decrease Δ = 0.05 and the mass of *m*(*h*_1_) increase the Δ. And the BPA will become *m*(*h*_1_) = 1 finally. Figure 5 shows the variation curve of the total uncertainty obtained by each measurement method.

Intuitively, BPA goes from *m*(Ω) = 1 to *m*(*h*_1_) = 1, which means it goes from completely unknown to certain. The total uncertainty should also be gradually reduced to 0. Figure 5 shows that all the four measurement methods can correctly reflect the changing trend of total uncertainty. However, *AU* and *AM* change slowly in the early stage and their sensitivity is not high. *TU*^*I*^ is a linear change, but its range of change is narrow. *AU*, *AM* and *ZU* showed nonlinear changes. Moreover, *ZU* has the widest variation range and higher variation speed.

**Figure 6:**
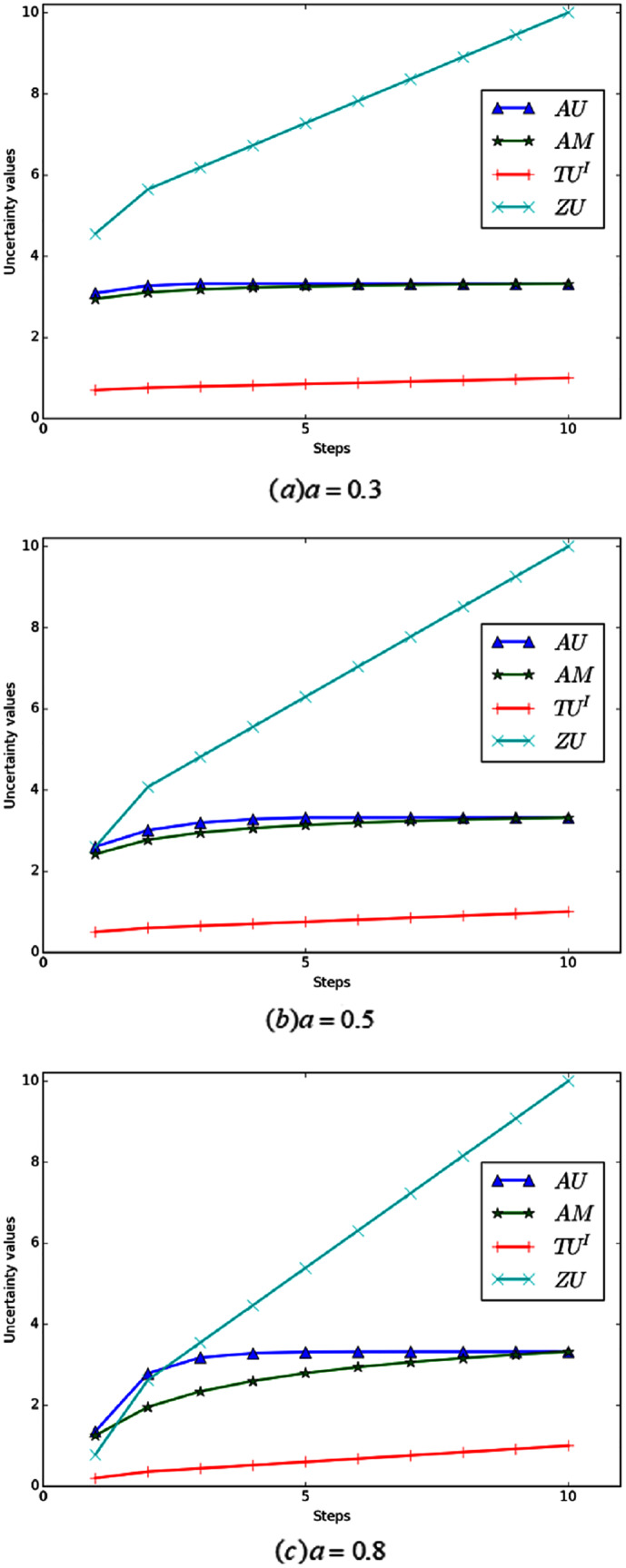
(A–C) The total uncertainty measures in Example 6.

**Example 6** Define that FOD $\Omega = \left\{ {{h_1},{h_2}, \cdots ,{h_{10}}} \right\}$, and the BPA is *m*(Ω) = 1 − *a*, *m*(*A*) = *a*. Now let the BPA changes according to certain rule. The initial condition is $A = \left\{ {{h_1}} \right\}$. At each step, adding an element *h*_*i*_ to *A*, and eventually *A* becomes the FOD Ω. Figure 6 shows the change curve of total uncertainty obtained by various measurement methods under different values of *a*.

Evidently, in the process of change, the total uncertainty is constantly increasing, because its non-specificity is constantly increasing. As shown in Fig. 6, When the value of *a* is small, the sensitivity of *AU* and *AM* is poor, especially in the latter part of the change process, and their values almost remain unchanged. When *a* increased to 0.8, the sensitivity of *AM* was improved, but the sensitivity of *AU* was still poor in the later period. Relatively speaking, the total uncertainty measured by *ZU* and *TU*^*I*^ always increases intuitively with the change of BPA. In addition, *ZU* ’s change is more obvious and its sensitivity is higher, which also reflects the rationality of *ZU*.

## Application in classification

In this section, we will apply the proposed *ZU* to two classification experiments to verify its effectiveness. This data set is derived from the UCI Machine Learning Repository.

### Experiment 1

In the iris dataset, there are three species and four attributes for classification. Each species contains 50 instances. In Wang, Zhang & Deng (2019), Wang et al. randomly selected 40 instances from each species to generate triangular fuzzy numbers, and the remaining 10 instances were used as a test set. Moreover, Wang et al. randomly selected an instance from the test set of Setosa (a) species to generate BPA. The results are shown in Table 2.

**Table 2:** BPAs of four attributes.

Attribute	*m*(*a*)	*m*(*b*)	*m*(*c*)	*m*(*a*,*b*)	*m*(*a*,*c*)	*m*(*b*,*c*)	*m*(*a*,*b*,*c*)
*SL*	0.3337	0.3165	0.2816	0.0307	0.0052	0.0272	0.0052
*SW*	0.3164	0.2501	0.2732	0.0304	0.0481	0.0515	0.0304
*PL*	0.6699	0.3258	0	0	0	0.0043	0
*PW*	0.6699	0.2778	0	0	0	0.0226	0

**Figure 7:**
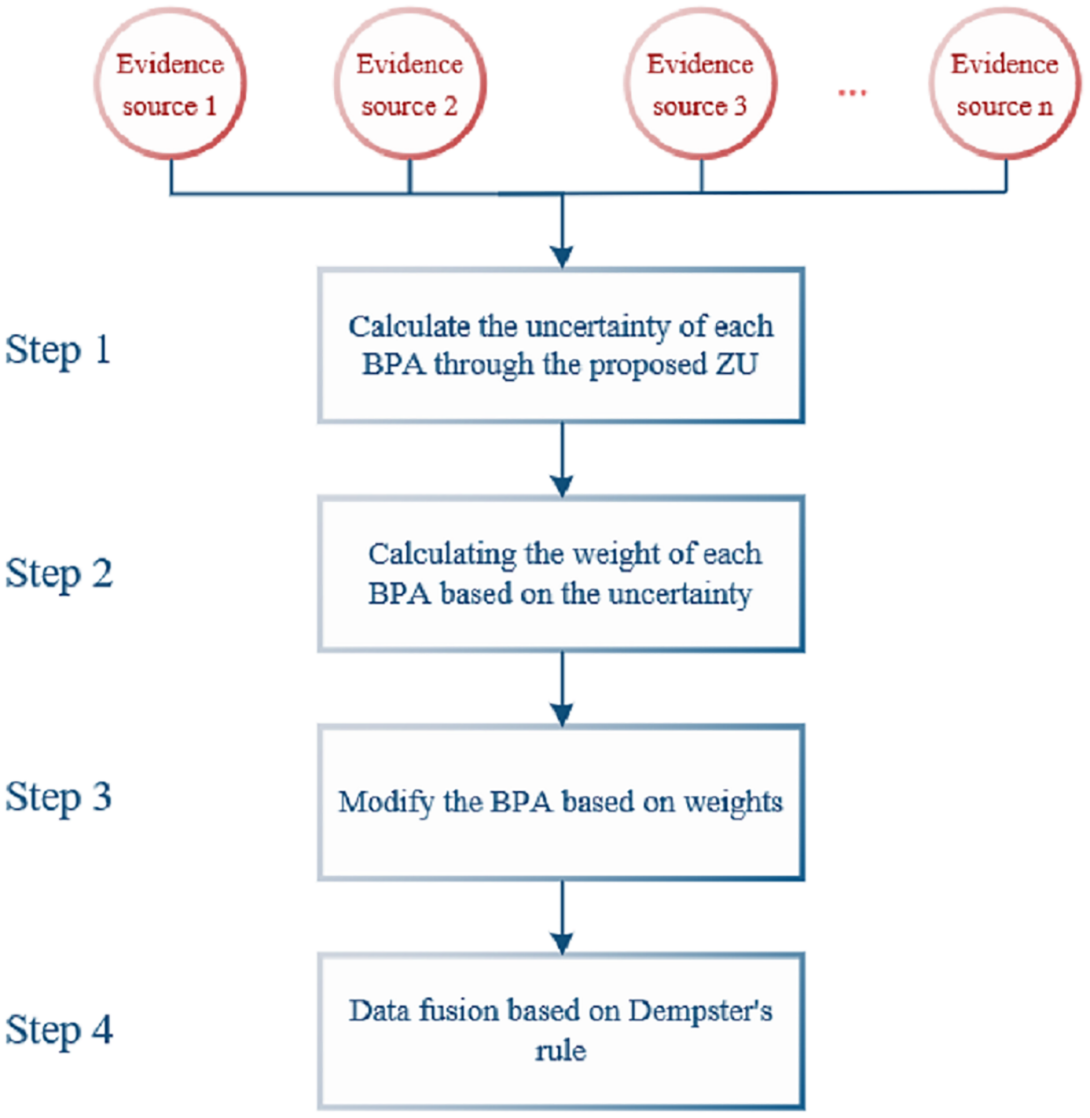
The flowchart of data fusion based on *ZU*.

In the following, we will show the specific application steps of *ZU* in the classification experiment, and the flow chart is shown in Fig. 7.

Under the above given conditions, the specific calculation steps in the application process of *ZU* are as follows:

Step 1: Calculate the uncertainty of each BPA. The calculation results are shown below.

$$\matrix{ {ZU\left( {{m_1}} \right) = \sum\limits_{i = 1}^3 \displaystyle{4 \over 3}\left( {\displaystyle{1 \over {{{(1 + d)}^2}}} - \displaystyle{1 \over 4}} \right) = 0.362} \hfill \cr {ZU\left( {{m_2}} \right) = 0.4418,ZU\left( {{m_3}} \right) = 0.2096,ZU\left( {{m_4}} \right) = 0.2047} \hfill \cr }$$

Step 2: Calculating the weight of each BPA based on the uncertainty.

In this paper, the weight definition method refers to the (Jiang et al., 2016). This method attaches great importance to uncertain and unknown information. The greater the uncertain degree calculated by the measure, the more uncertain information it contains, so the higher weight should be given. Therefore, the weight calculation method in this paper is as follows:

(12) $$w\left( {{m_i}} \right) = \displaystyle{{ZU\left( {{m_i}} \right)} \over {\sum\nolimits_{i = 1}^n ZU\left( {{m_i}} \right)}}$$

The calculation results of each weight are as follows:

$$\matrix{ {w\left( {{m_1}} \right) = 0.2972,w\left( {{m_2}} \right) = 0.3627} \hfill \cr {w\left( {{m_3}} \right) = 0.1721,w\left( {{m_4}} \right) = 0.2048} \hfill \cr }$$

Step 3: Modify the BPA based on weight factor. The modified BPA can be obtained by the following calculation method:

(13) $${m_w}(a) = \sum\limits_{i = 1}^4 {w_i}{m_i}(a).$$

The modified BPA is as follows:

$$\matrix{ {{m_w}(a) = 0.4468,{m_w}(b) = 0.2875,{m_w}(c) = 0.1828,} \hfill \cr {{m_w}(a,b) = 0.0201,{m_w}(a,c) = 0.019,} \hfill \cr {{m_w}(b,c) = 0.0313,{m_w}(\Omega ) = 0.0126.} \hfill \cr }$$

Step 4: Information fusion based on Dempster’s rule. According to Murphy’s evidence fusion strategy (Murphy, 2000), we combine the modified BPA (*n* − 1) times by the Dempster’s rule. And in this experiment, *n* = 4. So the fusion results are as follows:

$$\matrix{ {m(a) = {{\left( {{{\left( {{{\left( {{m_w} \oplus {m_w}} \right)}_1} \oplus {m_w}} \right)}_2} \oplus {m_w}} \right)}_3}(a) = 0.7592,} \hfill \cr {m(b) = 0.1841,m(c) = 0.043,} \hfill \cr {m(a,b) = m(a,c) = m(b,c) = m(\Omega ) = 0.} \hfill \cr }$$

According to the above results, Iris species Setosa (a) has the highest confidence, so the species is Setosa (a), which is consistent with the reality. Table 3 shows the comparison of classification results of different methods. All the three methods can correctly conclude that Setosa (a) is the target species. But compared with other methods, the proposed *ZU* has a great improvement in the accuracy of classification results. This reflects the effectiveness of the application of *ZU* classification.

**Table 3:** Results of Iris classification with different methods.

Methods	*m*(*a*)	*m*(*b*)	*m*(*c*)	*m*(Ω)
Yager rule (Yager, 1987)	0.5337	0.1484	0.0000	0.3180
Method in (Wang, Zhang & Deng, 2019)	0.6232	0.2671	0.1083	0.0000
Proposed *ZU*	0.7592	0.1841	0.0430	0.0000

### Experiment 2

In reference (Yuan & Deng, 2019), Yuan et al. used 120 samples from the iris dataset as the training set and the remaining 30 samples as the test set to generate BPA. And the evidence of SW characteristics is disturbed to produce conflicting evidence. In this paper, five groups of BPAs from Setosa (a) species generate by Yuan et al. are selected for the classification experiment. The BPAs of each sample is shown in Table 4.

**Table 4:** BPAs of four attributes of five samples.

	*SL*	*SW*	*PL*	*PW*
Sample 1	*m*(*a*) = 0.8650	*m*(*a*) = 0.0000	*m*(*a*) = 0.6486	*m*(*a*) = 0.7477
	*m*(*b*) = 0.0000	*m*(*b*) = 0.9000	*m*(*b*) = 0.0000	*m*(*b*) = 0.0000
	*m*(*a*,*b*) = 0.0821	*m*(*a*,*b*) = 0.0000	*m*(*a*,*b*) = 0.0000	*m*(*a*,*b*) = 0.0000
	*m*(*b*,*c*) = 0.0000	*m*(*b*,*c*) = 0.1000	*m*(*b*,*c*) = 0.0000	*m*(*b*,*c*) = 0.0000
	*m*(Ω) = 0.0529	*m*(Ω) = 0.0000	*m*(Ω) = 0.3514	*m*(Ω) = 0.2523
Sample 2	*m*(*a*) = 0.1356	*m*(*a*) = 0.0000	*m*(*a*) = 0.6486	*m*(*a*) = 0.7547
	*m*(*b*) = 0.0000	*m*(*b*) = 0.9000	*m*(*b*) = 0.0000	*m*(*b*) = 0.0000
	*m*(*a*,*b*) = 0.0000	*m*(*a*,*b*) = 0.0000	*m*(*a*,*b*) = 0.0000	*m*(*a*,*b*) = 0.0000
	*m*(*b*,*c*) = 0.0000	*m*(*b*,*c*) = 0.1000	*m*(*b*,*c*) = 0.0000	*m*(*b*,*c*) = 0.0000
	*m*(Ω) = 0.8644	*m*(Ω) = 0.0000	*m*(Ω) = 0.3514	*m*(Ω) = 0.2453
Sample 3	*m*(*a*) = 0.6780	*m*(*a*) = 0.0000	*m*(*a*) = 0.8649	*m*(*a*) = 0.7477
	*m*(*b*) = 0.0000	*m*(*b*) = 0.9000	*m*(*b*) = 0.0000	*m*(*b*) = 0.0000
	*m*(*a*,*b*) = 0.0000	*m*(*a*,*b*) = 0.0000	*m*(*a*,*b*) = 0.0000	*m*(*a*,*b*) = 0.0000
	*m*(*b*,*c*) = 0.0000	*m*(*b*,*c*) = 0.1000	*m*(*b*,*c*) = 0.0000	*m*(*b*,*c*) = 0.0000
	*m*(Ω) = 0.3220	*m*(*Ω*) = 0.0000	*m*(*Ω*) = 0.1351	*m*(*Ω*) = 0.2523
Sample 4	*m*(*a*) = 0.4068	*m*(*a*) = 0.0000	*m*(*a*) = 0.8649	*m*(*a*) = 0.7547
	*m*(*b*) = 0.0000	*m*(*b*) = 0.9000	*m*(*b*) = 0.0000	*m*(*b*) = 0.0000
	*m*(*a*,*b*) = 0.0000	*m*(*a*,*b*) = 0.0000	*m*(*a*,*b*) = 0.0000	*m*(*a*,*b*) = 0.0000
	*m*(*b*,*c*) = 0.0000	*m*(*b*,*c*) = 0.1000	*m*(*b*,*c*) = 0.0000	*m*(*b*,*c*) = 0.0000
	*m*(Ω) = 0.5932	*m*(Ω) = 0.0000	*m*(Ω) = 0.1351	*m*(Ω) = 0.2453
Sample 5	*m*(*a*) = 0.5253	*m*(*a*) = 0.0000	*m*(*a*) = 0.9143	*m*(*a*) = 0.7547
	*m*(*b*) = 0.0000	*m*(*b*) = 0.9000	*m*(*b*) = 0.0000	*m*(*b*) = 0.0000
	*m*(*a*,*b*) = 0.2887	*m*(*a*,*b*) = 0.0000	*m*(*a*,*b*) = 0.0000	*m*(*a*,*b*) = 0.0000
	*m*(*b*,*c*) = 0.0000	*m*(*b*,*c*) = 0.1000	*m*(*b*,*c*) = 0.0000	*m*(*b*,*c*) = 0.0000
	*m*(Ω) = 0.1860	*m*(Ω) = 0.0000	*m*(Ω) = 0.0857	*m*(Ω) = 0.2453

According to the application steps of *ZU* as shown in Fig. 7, each sample is calculated and its classification results are obtained. Table 5 shows the comparison of classification results of the methods proposed by *ZU* and Yuan et al. The classification results of the two methods can correctly conclude that the species is species Setosa (a). However, compared with method in Yuan & Deng (2019), the proposed *ZU* had a higher classification accuracy, and the accuracy was improved significantly. Therefore, it could be explained that the problem of conflicting data could be effectively dealt with by the proposed *ZU* and more information could be obtained in the framework of D–S theory. The classification results show the validity and rationality of this method.

**Table 5:** Results of different methods for five samples.

	Methods	*m*(*a*)	*m*(*b*)	*m*(*c*)	*m*(*a*,*b*)	*m*(*a*,*c*)	*m*(*b*,*c*)	*m*(*Ω*)
Sample 1	Method in (Yuan & Deng, 2019)	0.9450	0.0088	0.0000	0.0047	0.0000	0.0008	0.0406
	Proposed *ZU*	0.9796	0.0123	0.0000	0.0010	0.0000	0.0008	0.0053
Sample 2	Method in (Yuan & Deng, 2019)	0.7221	0.0312	0.0000	0.0000	0.0000	0.0033	0.2435
	Proposed *ZU*	0.9472	0.0574	0.0000	0.0546	0.0000	0.0046	0.1595
Sample 3	Method in (Yuan & Deng, 2019)	0.9562	0.0074	0.0000	0.0000	0.0000	0.0080	0.0357
	Proposed *ZU*	0.9840	0.0103	0.0000	0.0000	0.0007	0.0000	0.0046
Sample 4	Method in (Yuan & Deng, 2019)	0.8100	0.0207	0.0000	0.0000	0.0000	0.0022	0.1671
	Proposed *ZU*	0.9340	0.0264	0.0000	0.0000	0.0000	0.0023	0.0371
Sample 5	Method in (Yuan & Deng, 2019)	0.8475	0.0175	0.0000	0.0645	0.0000	0.0011	0.0695
	Proposed *ZU*	0.9595	0.0183	0.0000	0.0130	0.0000	0.0003	0.0014

## Conclusion

In this paper, a new uncertainty measurement method is proposed based on the belief interval uncertainty measure. This method can not only avoid the problem of information loss caused by framework transformation, but also solve the defects of the old measure. In the examples of uncertainty measurement, the new measure *ZU* shows a good effect. Compared with the old measures, *ZU* is more sensitive to the change of BPA and can better measure the uncertainty of each BPA. In addition, we propose a data fusion method based on *ZU*, which takes the uncertainty of each evidence as the weight, and then modifies the evidence, so as to reduce the conflict or unreliability between the evidence. Then, Dempster’s rule is used for data fusion. Through the experiments, we can find that the proposed *ZU* has higher classification accuracy. This reflects the effectiveness and rationality of *ZU*.

In general, *ZU* can effectively deal with the problem of conflicting data and obtain more effective information from the framework of D–S theory so as to obtain more accurate information measure and fusion results. However, it should be noted that *ZU* still has some open issues for studying. The proposed *ZU* can’t be applied to the open-world hypothesis (Smets, 1990; Deng, 2015; Tang, Wu & Liu, 2021) of increasing uncertain information sources. And we found that *ZU* maybe ineffective in some conditions. It is assumed that the total uncertainty obtained by *ZU* will fail when the belief intervals of singleton elements obtained by the two BPA groups are the same.

Define that FOD $\Omega = \left\{ {{h_1},{h_2},{h_3}, {h_4}} \right\}$, two BPAs are given as:

$$\matrix{ {} \hfill  {\left\{ {\matrix{ {{m_1}\left( {{h_1}},{h_{2}} \right) = {m_1}\left( {{h_3}},h_{4} \right) =  0.2} \hfill \cr {{m_1}\left( {{h_1},{h_2},{h_3}} \right) = {m_1}\left( {{h_1},{h_2},{h_4}} \right) = {m_1}\left( {{h_1},{h_3},{h_4}} \right)= {m_1}\left( {{h_2},{h_3},{h_4}} \right) = 0.1} \hfill \cr {{m_1}\left( \Omega \right) = 0.2.} \hfill \cr } } \right.} \hfill \cr {} \hfill  {\left\{ {\matrix{ {{m_2}\left( {{h_1}},{{h_2}} \right) = {m_2}\left( {{h_3}},{{h_4}} \right) = 0.1} \hfill \cr {{m_2}\left( {{h_1},{h_2},{h_3}} \right) = {m_2}\left( {{h_1},{h_2},{h_4}} \right) = {m_2}\left( {{h_1},{h_3},{h_4}} \right)= {m_2}\left( {{h_2},{h_3},{h_4}} \right) = 0.2.} \hfill \cr } } \right.} \hfill \cr }$$

After calculation, the belief interval of the singletons obtained for both BPA groups is $\left[ {Bel\left( {{h_i}} \right),Pl\left( {{h_i}} \right)} \right] = \left[ {0,0.7} \right],i = 1,2,3,4$. So *ZU*(*m*) gives the same total uncertainty, which is $ZU\left( {{m_1}} \right) = ZU\left( {{m_2}} \right) = 1.822$. The reason for this may be that using belief intervals for singletons alone may not fully specify a set of BPA. Therefore, in the following time, we will try to solve this problem by adding the belief interval of multiple elements into the calculation formula of ZU and we will continue to study how to extend ZU to the open-world hypothesis.

## Supplemental Information

10.7717/peerj-cs.710/supp-1 Supplemental Information 1 The Iris dataset for the experiment.

10.7717/peerj-cs.710/supp-2 Supplemental Information 2 The code for each experiment.

## Additional Information and Declarations

### Data Availability

The following information was supplied regarding data availability: The raw data and code are available in the Supplemental Files.
