# Supplementary figures and images for: An improved measure for belief structure in the evidence theory
Source: PeerJ Comput Sci. 2021 Sep 24;7:e710. doi: 10.7717/peerj-cs.710 (PMC8507476; doi:10.7717/peerj-cs.710)

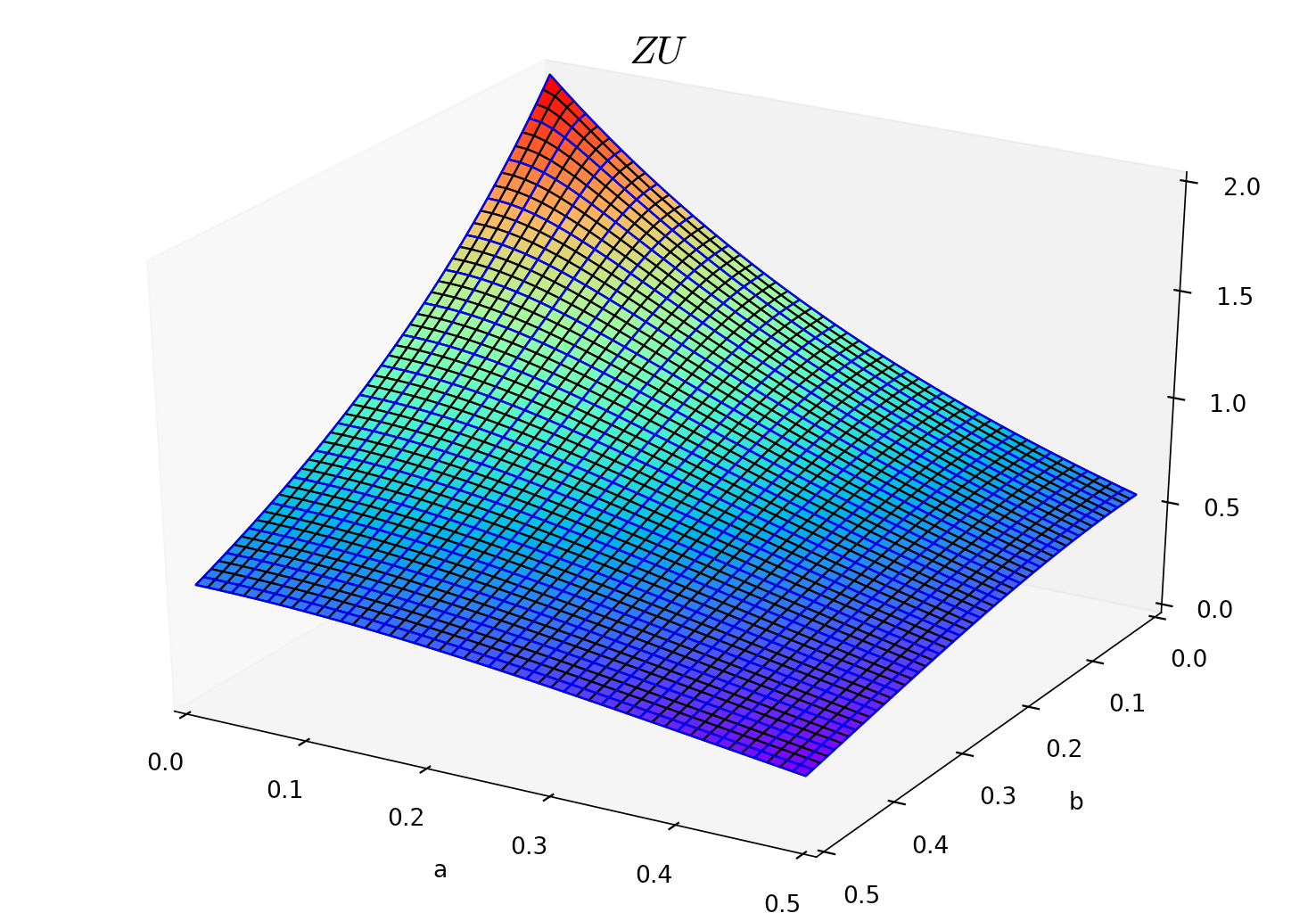

Supplement: Supplemental Information 2 [file peerj-cs-07-710-s002.zip › PeerJ-computer code/Example3_1/$ZU$.png]

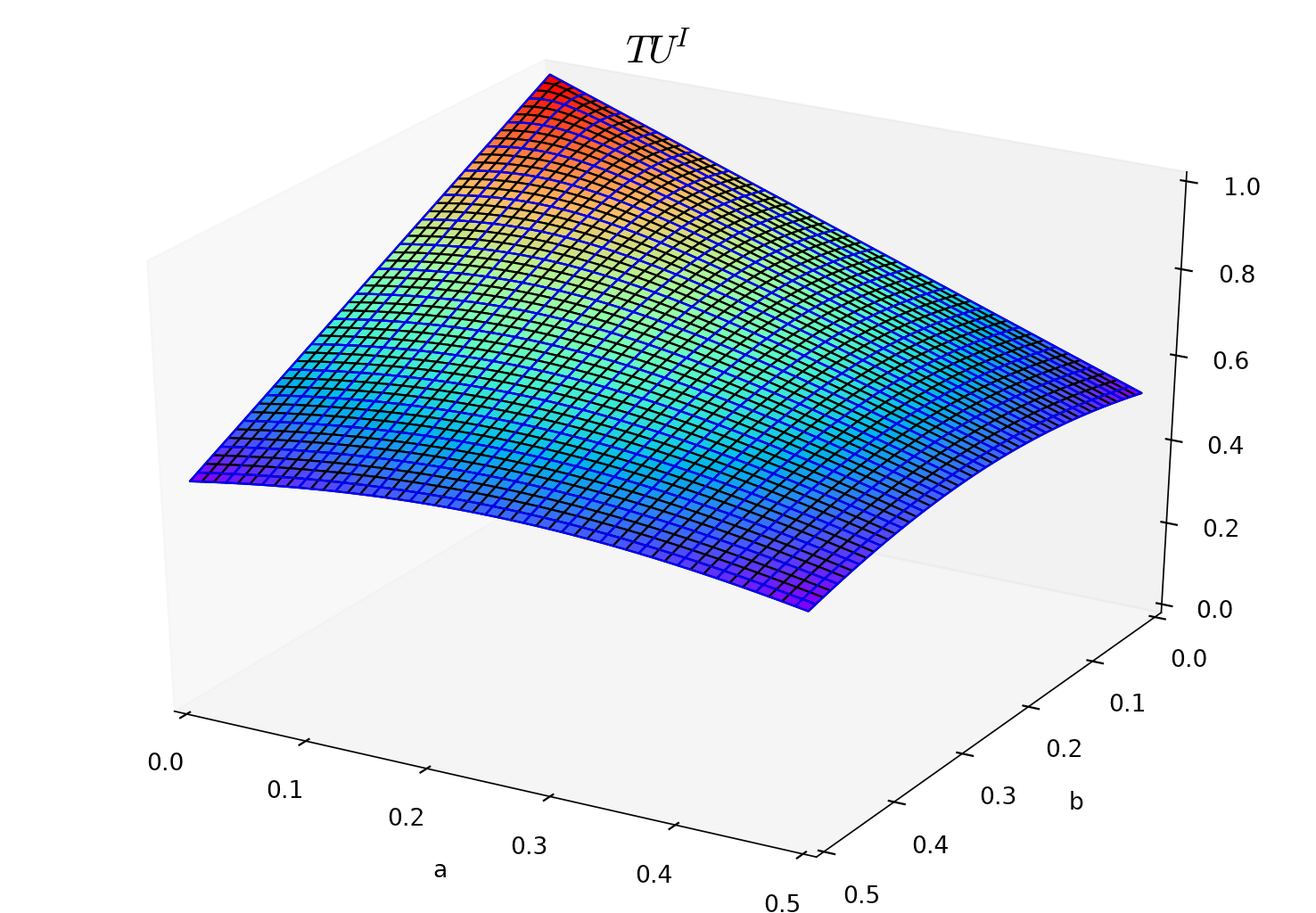

Supplement: Supplemental Information 2 [file peerj-cs-07-710-s002.zip › PeerJ-computer code/Example3_1/${TU}^{I}$.png]

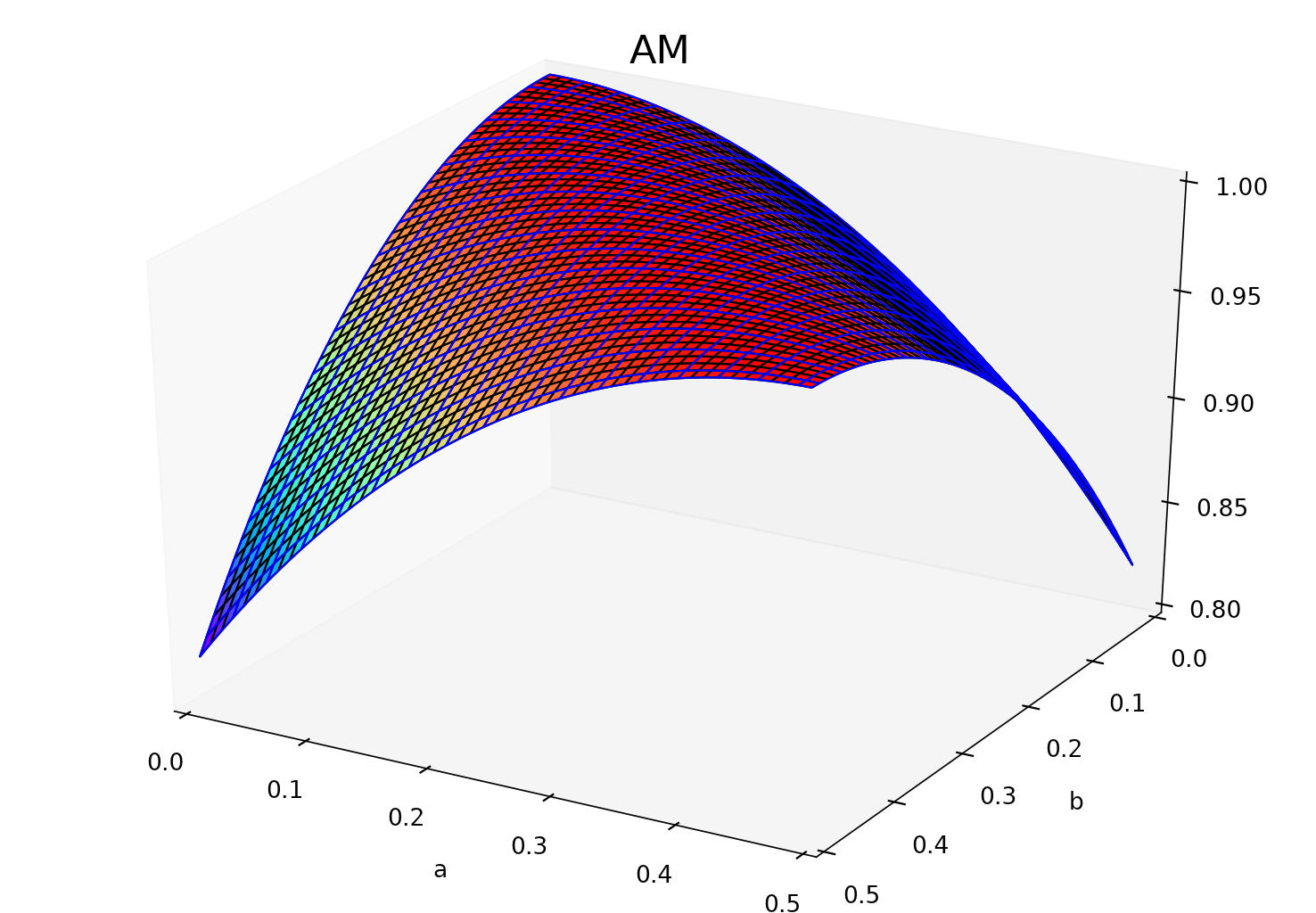

Supplement: Supplemental Information 2 [file peerj-cs-07-710-s002.zip › PeerJ-computer code/Example3_1/AM.png]

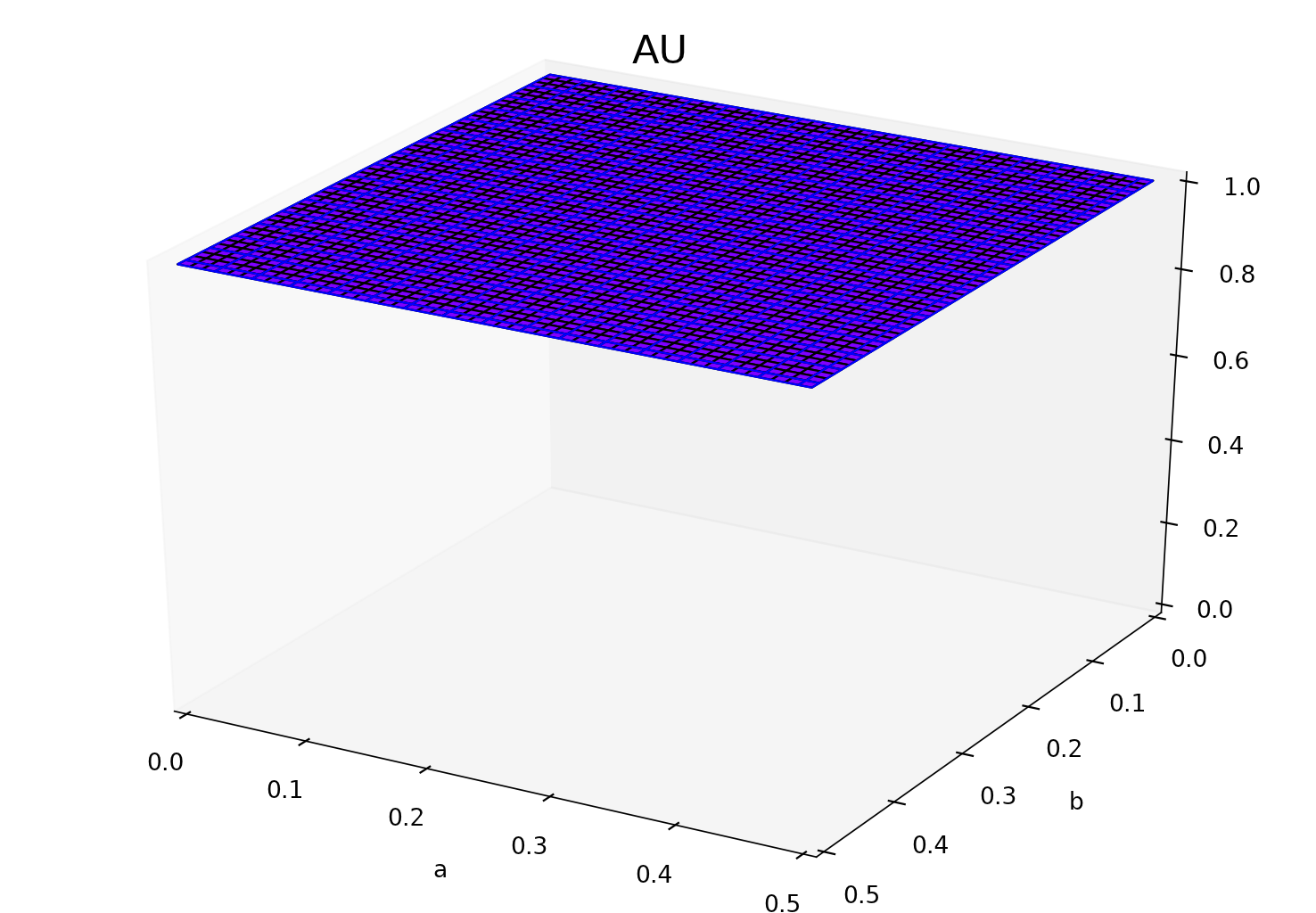

Supplement: Supplemental Information 2 [file peerj-cs-07-710-s002.zip › PeerJ-computer code/Example3_1/AU.png]

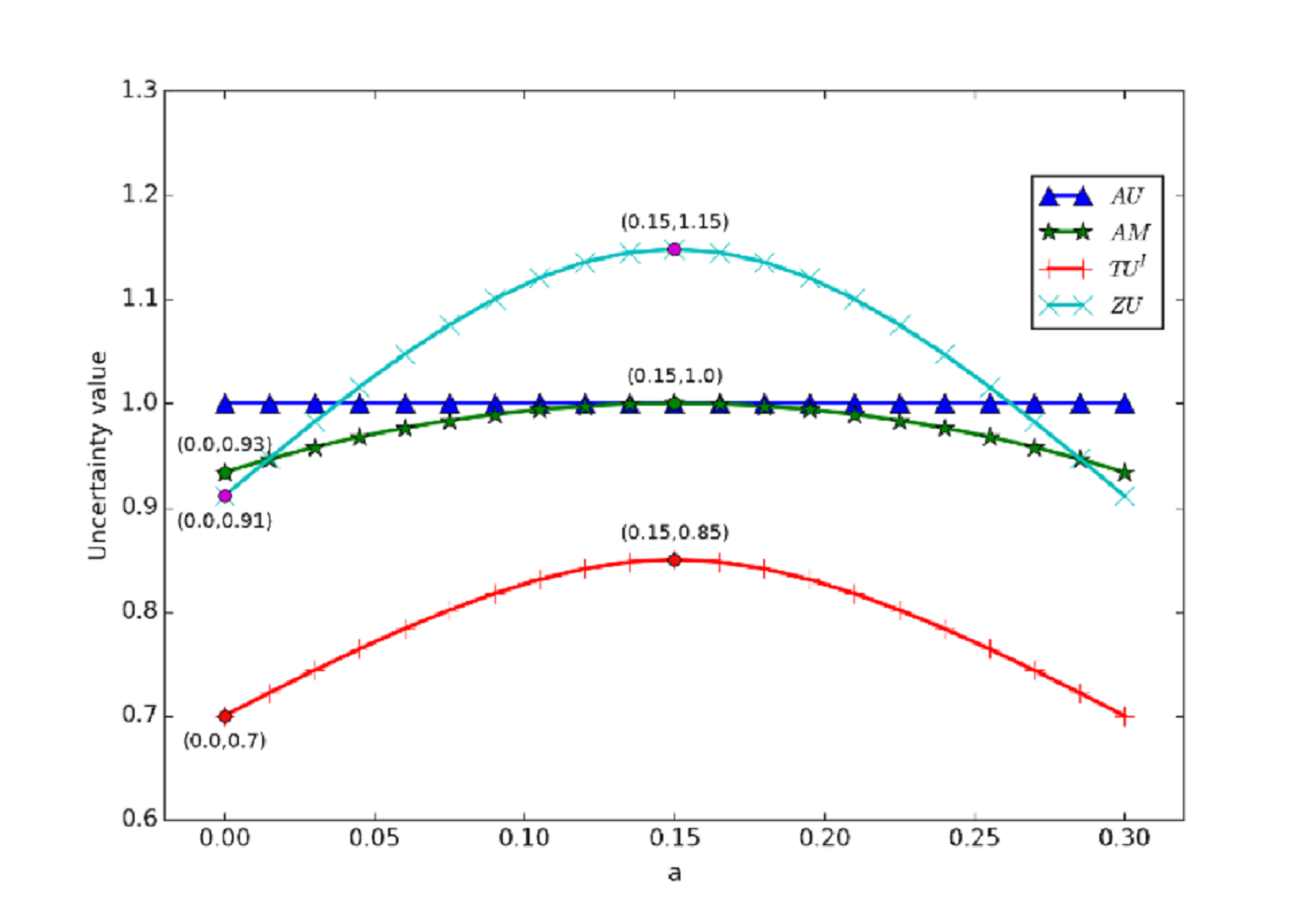

Supplement: Supplemental Information 2 [file peerj-cs-07-710-s002.zip › PeerJ-computer code/Example3_2/EX3-2.png]

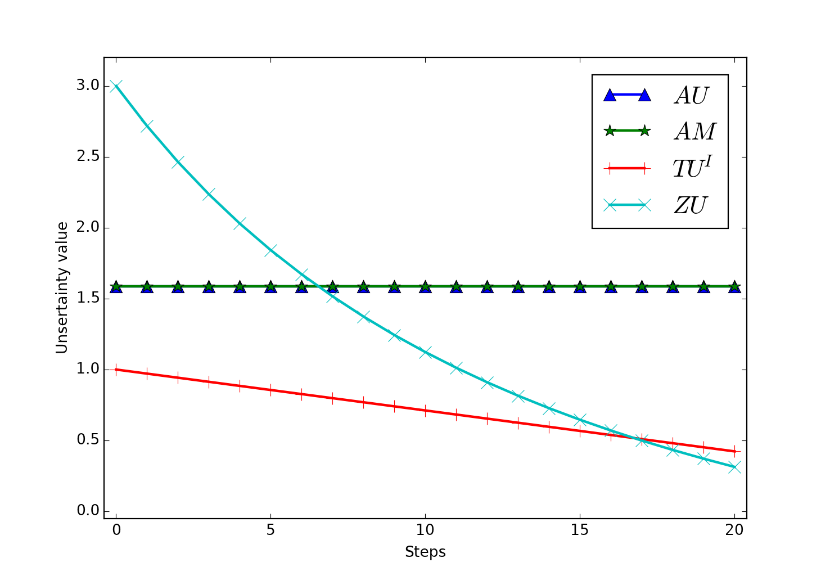

Supplement: Supplemental Information 2 [file peerj-cs-07-710-s002.zip › PeerJ-computer code/Example4/EX4-1.png]

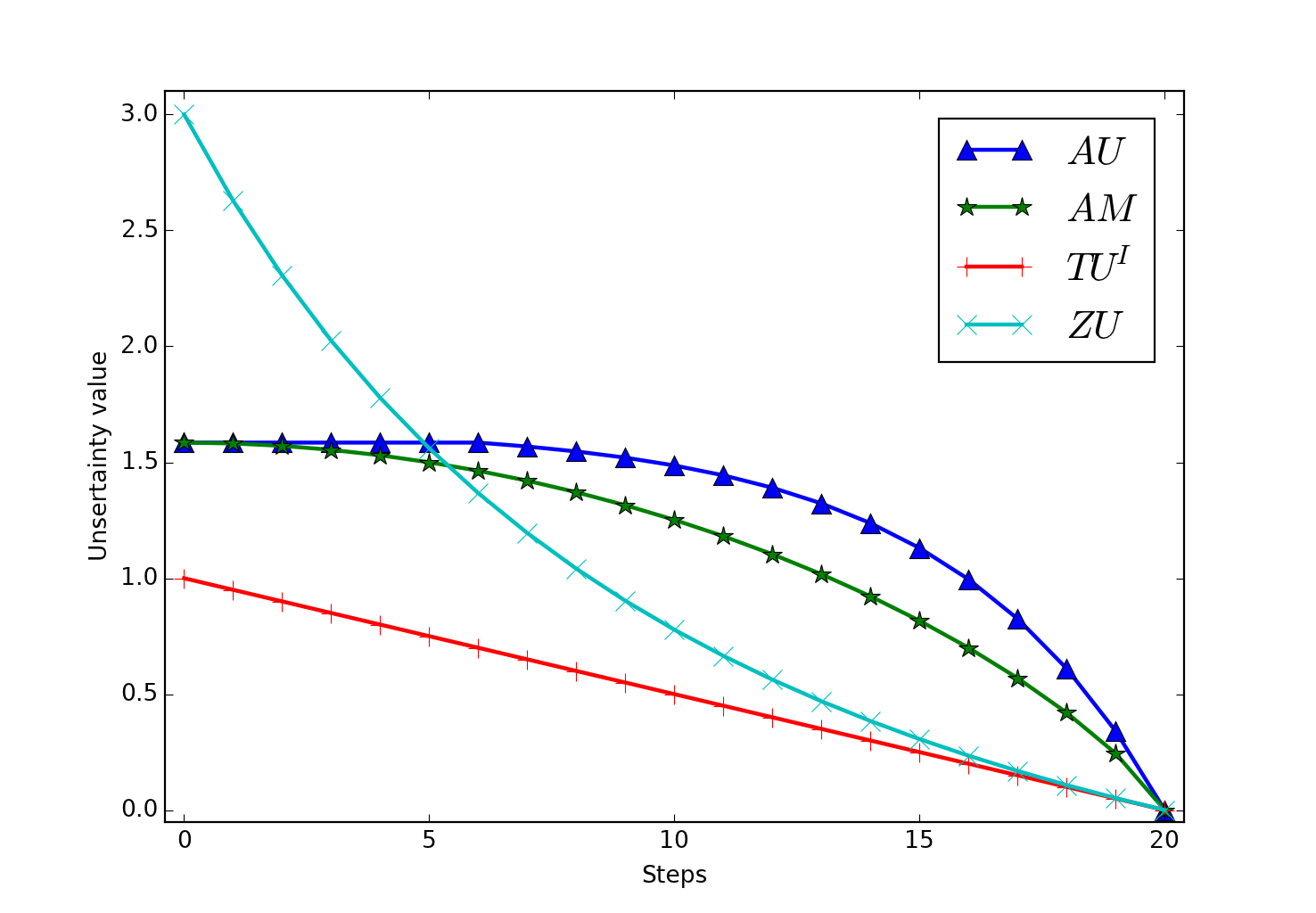

Supplement: Supplemental Information 2 [file peerj-cs-07-710-s002.zip › PeerJ-computer code/Example5/EX6-1.png]

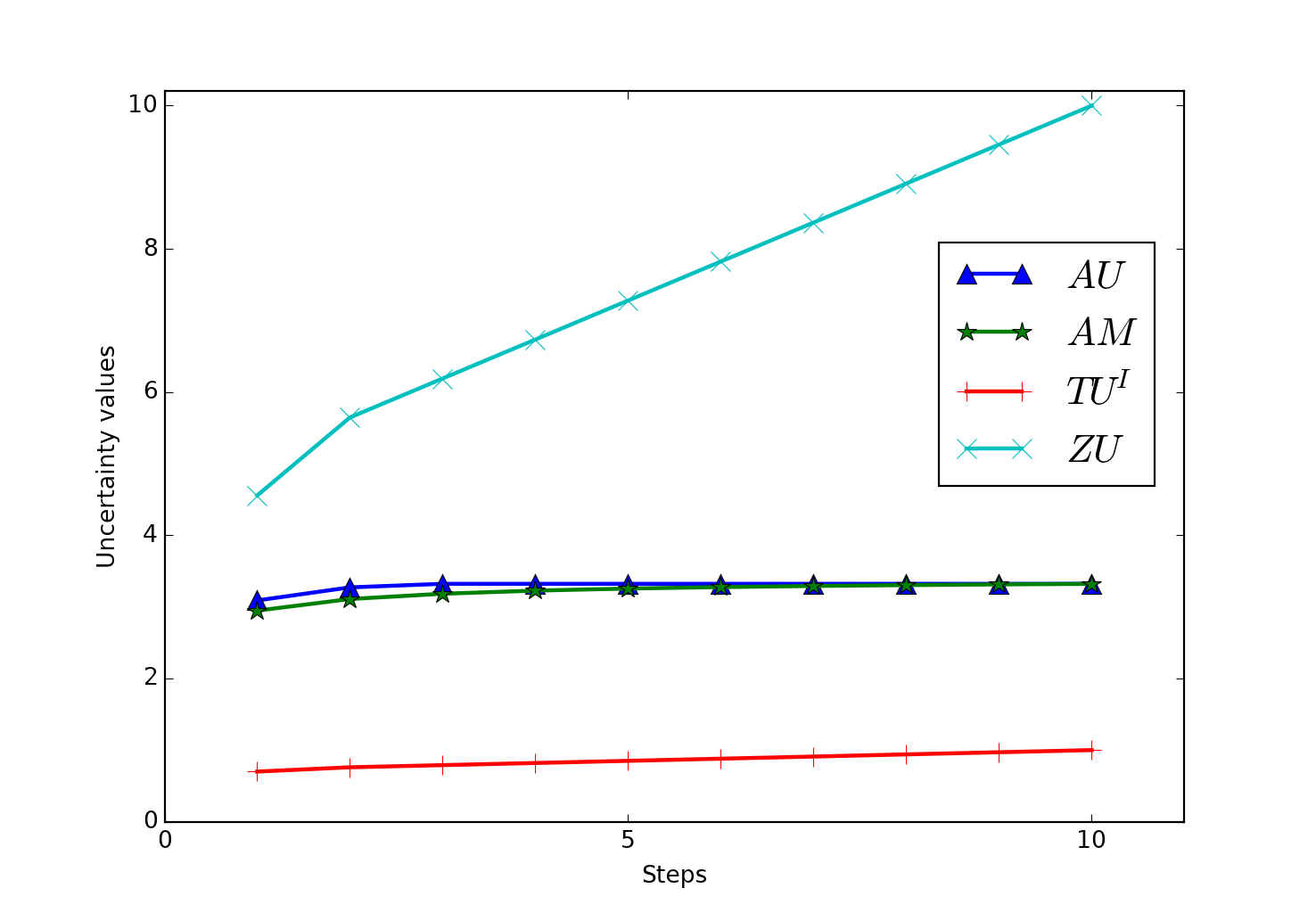

Supplement: Supplemental Information 2 [file peerj-cs-07-710-s002.zip › PeerJ-computer code/Example6/E8-1.png]

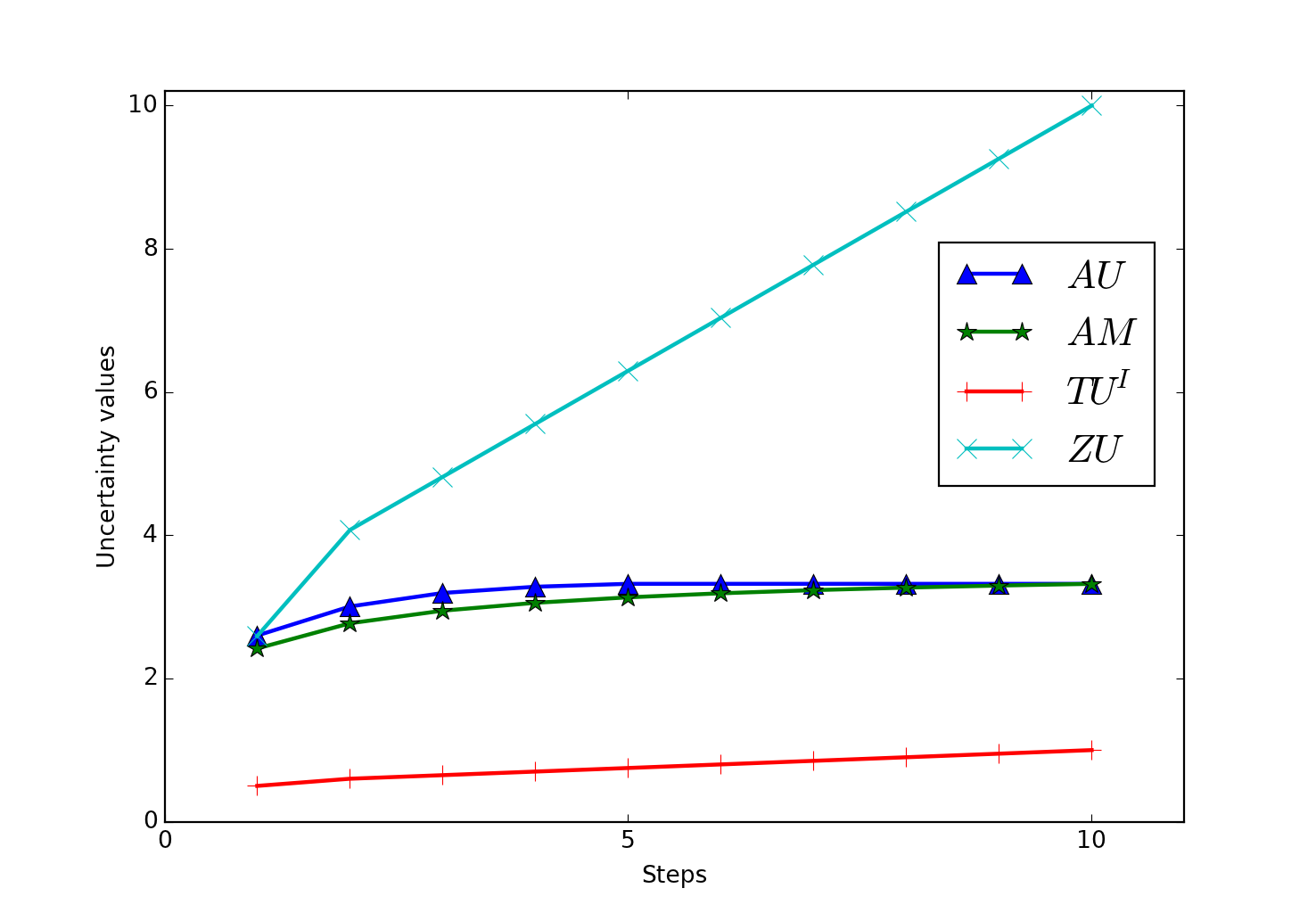

Supplement: Supplemental Information 2 [file peerj-cs-07-710-s002.zip › PeerJ-computer code/Example6/E8-2.png]

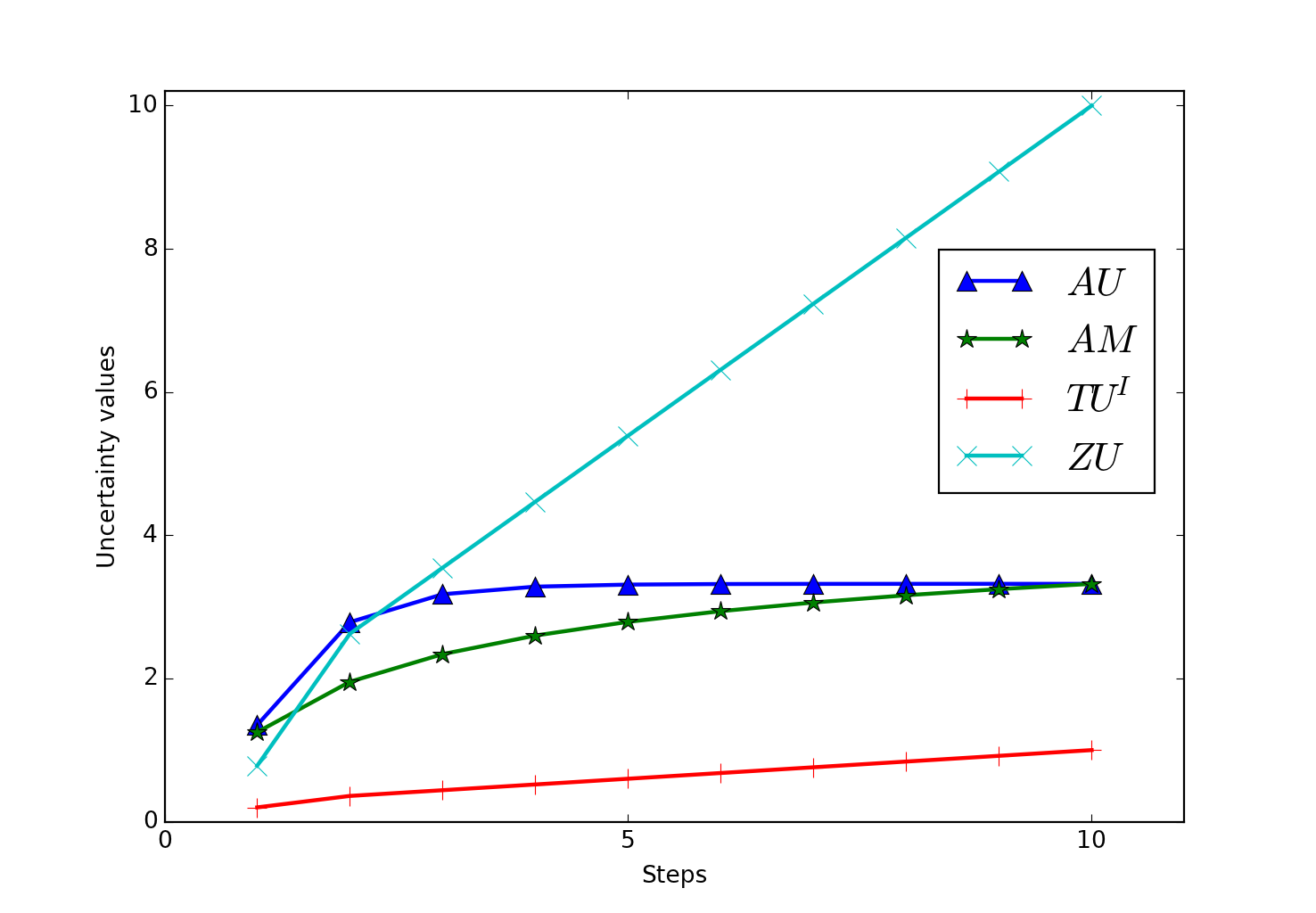

Supplement: Supplemental Information 2 [file peerj-cs-07-710-s002.zip › PeerJ-computer code/Example6/E8-3.png]
